# Cerebrospinal fluid proteome profiling across the Alzheimer’s disease continuum: a step towards solving the equation for ‘X’

**DOI:** 10.1186/s13024-025-00841-0

**Published:** 2025-05-06

**Authors:** Sophia Weiner, Mathias Sauer, Laia Montoliu-Gaya, Andrea L. Benedet, Nicholas J. Ashton, Fernando Gonzalez-Ortiz, Joel Simrén, Nesrine Rahmouni, Cecile Tissot, Joseph Therriault, Stijn Servaes, Jenna Stevenson, Ville Leinonen, Tuomas Rauramaa, Mikko Hiltunen, Pedro Rosa-Neto, Kaj Blennow, Henrik Zetterberg, Johan Gobom

**Affiliations:** 1 Department of Psychiatry and Neurochemistry, Institute of Neuroscience and Physiology, University of Gothenburg, Mölndal, Sweden; 2 Banner Alzheimer’s Institute and University of Arizona, Phoenix, AZ USA; 3 Clinical Neurochemistry Lab, Institute of Neuroscience and Physiology, Sahlgrenska University Hospital, Mölndal, Sweden; 4 McGill University Research Centre for Studies in Aging, Montreal, QC Canada; 5 Department of Neurosurgery, NeuroCenter, Kuopio University Hospital and Neurosurgery, Institute of Clinical Medicine, University of Eastern Finland, Kuopio, Finland; 6 Department of Pathology, Kuopio University Hospital and Institute of Clinical Medicine-Pathology, University of Eastern Finland, Kuopio, Finland; 7 Institute of Biomedicine, University of Eastern Finland, Kuopio, Finland; 8 Translational Neuroimaging Laboratory, McGill University Research Centre for Studies in Aging, Department of Neurology and Neurosurgery, Psychiatry and Pharmacology and Therapeutics, McGill University, Montreal, Canada; 9 Paris Brain Institute, ICM, Pitié-Salpêtrière Hospital, Sorbonne University, Paris, France; 10 Neurodegenerative Disorder Research Center, Division of Life Sciences and Medicine, and Department of Neurology, Institute On Aging and Brain Disorders, University of Science and Technology of China and First Affiliated Hospital of USTC, Hefei, People’s Republic of China; 11 Department of Neurodegenerative Disease, UCL Institute of Neurology, London, UK; 12 UK Dementia Research Institute, UCL, London, UK; 13 Hong Kong Center for Neurodegenerative Diseases, Clear Water Bay, Hong Kong, China; 14 Wisconsin Alzheimer’s Disease Research Center, University of Wisconsin School of Medicine and Public Health, University of Wisconsin-Madison, Madison, WI USA

**Keywords:** Cerebrospinal fluid, Biomarkers, Alzheimer’s disease, Mass spectrometry, Tandem mass tag

## Abstract

**Background:**

While the temporal profile of amyloid (Aβ) and tau cerebrospinal fluid (CSF) biomarkers along the Alzheimer’s disease (AD) continuum is well-studied, chronological changes of CSF proteins reflecting other disease-relevant processes, denoted ‘X’ in the ATX(N) framework, remain poorly understood.

**Methods:**

Using an untargeted mass spectrometric approach termed tandem mass tag (TMT), we quantified over 1500 CSF proteins across the AD continuum in three independent cohorts, finely staged by Aβ/tau positron emission tomography (PET), fluid biomarkers, or brain biopsy. Weighted protein co-expression network analysis identified clusters of proteins robustly correlating in all three cohorts which sequentially changed with AD progression. Obtained protein clusters were correlated with fluid biomarker measurements (phosphorylated tau (p-tau) species including p-tau_181_, p-tau_217_, and p-tau_205_, as well as Aβ), Aβ/tau PET imaging, and clinical parameters to discern disease-relevant clusters which were modelled across the AD continuum.

**Results:**

Neurodegeneration-related proteins (e.g*.*, 14–3-3 proteins, PPIA), derived from different brain cell types, strongly correlated with fluid as well as imaging biomarkers and increased early in the AD continuum. Among them, the proteins SMOC1 and CNN3 were highly associated with Aβ pathology, while the 14–3-3 proteins YWHAZ and YWHAE as well as PPIA demonstrated a strong association with both Aβ and tau pathology as indexed by PET. Endo-lysosomal proteins (e.g., HEXB, TPP1, SIAE) increased early in abundance alongside neurodegeneration-related proteins, and were followed by increases in metabolic proteins such as ALDOA, MDH1, and GOT1 at the mild cognitive impairment (MCI) stage. Finally, later AD stages were characterized by decreases in synaptic/membrane proteins (e.g., NPTX2).

**Conclusions:**

Our study identified proxies of Aβ and tau pathology, indexed by PET, (SMOC1, YWHAE, CNN3) and highlighted the dynamic fluctuations of the CSF proteome over the disease course, identifying candidate biomarkers for disease staging beyond Aβ and tau.

**Supplementary Information:**

The online version contains supplementary material available at 10.1186/s13024-025-00841-0.

## Background

An ageing global population has reshaped our medical landscape, transforming Alzheimer’s disease (AD)—the most prevalent age-related neurodegenerative disorder—into a global health challenge [1]. In the clinical continuum of AD, patients often first report subjective cognitive deficits before progressing to a measurable mild cognitive impairment (MCI) and eventually to AD with dementia [2]. Pathological changes in the brain, however, appear decades before the first symptoms manifest [3], emphasizing the long preclinical phase of the disease.

On a molecular level, one of the earliest known changes to occur in AD is aggregation of the amyloid-β (Aβ) 1–42 peptide, leading to extracellular Aβ plaque formation in the brain [4]. Amyloid pathology is closely followed by tau hyperphosphorylation, with aggregation and deposition into intracellular neurofibrillary tangles (NFT) [4, 5], and a successive spread of the pathology and neurodegeneration throughout the brain. Although Aβ plaque formation precedes tau pathology, it has been shown that NFT load is, in fact, more closely linked to cognition and disease progression [6]. The spread of tau pathology throughout the brain commonly follows a distinct topographic pattern, which according to the Braak staging system [7] begins in the medial temporal regions (Braak I-II) and progresses to the limbic regions (Braak III-IV) before reaching the neocortex (Braak V-VI). Both Aβ and tau pathology can be visualized in vivo by positron emission tomography (PET) with tracers targeting Aβ plaques or NFTs, respectively [8]. Identification and measurement of both pathological hallmarks through imaging and fluid biomarkers has caused a shift away from a clinical definition of AD towards a biological conceptualization using the AT(N) research framework, which categorizes individuals based on biomarker evidence for Aβ (A), tau (T), and neurodegeneration (N) [9].

While the AT(N) framework provides a foundation for defining AD through its core pathologies, numerous other processes are affected in the disease, such as synaptic signalling, metabolism, lysosomal function, immune response, repair and regeneration [10–12]. Hence, the Alzheimer’s Association workgroup has recognized the need for incorporating new biomarker categories beyond AT(N), termed ATX(N), for disease diagnosis and staging, with ‘X’ denoting new unspecified biomarker categories like the recently added inflammation (I) or vascular brain injury (V) [13]. Many such processes are reflected in the heterogenous protein profile of cerebrospinal fluid (CSF) [14, 15]. CSF proteins could thus be used to monitor relevant pathophysiological processes occurring over the disease course, staging AD patients or even indexing molecular AD subtypes [16]. In addition, they can serve as more cost-effective proxies for PET imaging to monitor disease progression, while others may be targets for therapeutic intervention. Unfortunately, little is known about the temporal progression of CSF proteins reflecting disease-associated processes beyond Aβ/tau and their potential to stage AD patients. Recent large-scale proteomic studies in autosomal dominant and sporadic AD, however, have shown promise in identifying CSF protein alterations along the disease trajectory [17, 18].

Thus, attempting to ‘solve the equation for X’, we mapped CSF proteomic changes across the AD continuum by performing unbiased mass spectrometric proteomics analysis, using the tandem mass tag (TMT) technique, of the Translational Biomarkers in Aging and Dementia (TRIAD) cohort, consisting of patients at different stages of AD as well as cognitively unimpaired (CU) controls. The proteomics data were correlated with Aβ and tau PET data, cognitive test scores, and CSF p-tau profile. Further, changes in protein abundances were assessed across disease stages. The findings in the TRIAD discovery cohort were validated using data from two separate AD cohorts, in which participants were staged by fluid biomarkers or in vivo cortical brain biopsy.

## Methods

### The Translational Biomarkers in Aging and Dementia (TRIAD) cohort

The discovery proteomics analysis included 134 CSF samples from the TRIAD cohort. TRIAD includes participants across the whole AD spectrum as well as cognitively unimpaired controls. Patients have been profiled by clinical and neuropsychological assessments as well as by fluid and imaging biomarkers. Diagnosis of AD dementia followed the National Institute on Aging and the Alzheimer’s Association criteria for probable Alzheimer’s disease [19], with a Clinical Dementia Rating (CDR) greater than or equal to 0.5. MCI patients had a CDR of 0.5, reflecting subjective as well as objective memory impairments but preserved activities of daily living, while CU individuals had a CDR of 0. Study participants were further characterized by Aβ PET with [^18^F]AZD4694 and tau PET with [^18^F]MK6240, allowing in vivo detection and quantification of Aβ and tau pathology. The Aβ PET Standarized Uptake Value Ratio (SUVR) positivity threshold was set at 1.55 while tau PET positivity was assumed for SUVR values greater than 1.24 in accordance with a previous study [20]. Braak stages were derived from the neuropathologically determined brain regions as proposed by Braak [21]: Braak I (transentorhinal), Braak II (entorhinal/hippocampus), Braak III (amygdala, parahippocampal/fusiform/lingual gyrus), Braak IV (insula, inferior temporal, lateral temporal, posterior cingulate and inferior parietal), Braak V (orbitofrontal, inferior frontal, superior frontal, rostro medial frontal, anterior cingulate, superior temporal, supramarginal gyrus, superior parietal, precuneus, Cuneus, lateral occipital), Braak VI (precentral, pericalcarine, paracentral, postcentral) [22]. Each participant's Braak stages were determined hierarchically, signifying that the next stage could only be reached if the previous Braak stage was deemed positive; otherwise, the individual was classified as Braak discordant. The CSF core AD biomarkers Aβ_1-42_, Aβ_1-40_, p-tau_181_ and t-tau were measured using immunoassays (Lumipulse G, FujiRebio), and a mass spectrometric panel assay was employed to measure specific phosphorylated tau forms (CSF p-tau_181_, p-tau_199_, p-tau_202_, p-tau_205_, p-tau_217_, p-tau_231_, and p-tau_396_) [23].

### European Medical Information Framework for Alzheimer’s Disease (EMIF-AD) cohort

Data from a previously published proteomics study [24] of 467 CSF samples from the prospective, multicentred EMIF-AD cohort [25] were used for validation of the discovery proteomics findings. Inclusion criteria were having normal cognition or a diagnosis of MCI or AD-type dementia at baseline, age above 50 years, known Aβ status, as well as availability of cognitive test results. Participants were characterized by clinical assessment of dementia according to international consensus criteria, and by CSF Aβ_1-42_, t-tau and p-tau_181_ (INNOTEST ELISA) [25]. Aβ status was based on the CSF Aβ_42/40_ ratio with < 0.061 used as the cut-off for positivity. CSF p-tau positivity was not evaluated as analyses were performed at different sites and no harmonized measurements were available.

### Study population of the Kuopio idiopathic normal pressure hydrocephalus (iNPH) cohort

Proteomics data of the iNPH cohort were obtained from a previously published study [26]. The cohort consisted of lumbar CSF samples (acquired during CSF tap test) from patients with adult hydrocephalus, referred to Kuopio University Hospital (KUH) 2013–2021. All patients underwent shunt surgery during which frontal cortical biopsies (~ 2 mm in diameter and 3–10 mm in length) were obtained at the site where the ventricular catheter would penetrate the brain (~ 3 cm from the midline and anterior to the coronal suture). Aβ plaques and tau tangles in brain samples were stained using 6F3D and AT8 antibodies, evaluated by a neuropathologist, and graded semi-quantitatively using light microscopy. Description of core CSF biomarker measurements alongside cohort details can be found in [26–28].

### Sample preparation for TMT

For protein reduction, Tris(2-carboxyethyl)phosphine (TCEP) in sodium deoxycholate (DOC) and triethylammonium bicarbonate (TEAB) was added to each CSF sample (50 µL) to a final concentration of TCEP: 5 mM, DOC: 1%, TEAB: 100 mM. Samples were heated at 55 °C for one hour, equilibrated to room temperature and carbamidomethylation was performed by the addition of iodoacetamide to a concentration of 10 mM. Following incubation in the dark for 30 min, Trypsin (100 µg per vial; Promega) was added to each study sample (2.6 µg trypsin/50 µL CSF) for overnight incubation at 37 °C. The next day, TMTpro reagents (TMT 16plex, 5 mg; Thermo Fisher) were equilibrated to room temperature, reconstituted in 250 µL acetonitrile (ACN) and 10 µL were added to each sample for peptide labeling. The TMT labeling reaction commenced for one hour under constant shaking and was subsequently quenched by the addition of 6 µL 5% hydroxylamine solution (30 min incubation). Labeled samples were then combined into their corresponding TMT sets, with each set consisting of 15 study samples as well as one CSF pool sample (pooled CSF from all samples) for batch evaluation and quality control assessment. TMT sets were diluted with 0.1% trifluoroacetic acid (TFA) and acidified with hydrochloric acid (HCl) to precipitate DOC. The DOC precipitate was spun down at 4000 g for 15 min at 4 °C and the resulting supernatant was subjected to solid phase extraction (SPE). SPE was performed with reversed-phase C_18_ cartridges (Sep-Pak C18 light) operated under a vacuum manifold. Cartridges were first washed 1000 µL 0.1% TFA, 80% ACN and then twice equilibrated with 1000 µL 0.1% TFA. TMT sets were pipetted onto the column, washed with 2 × 1000 µL 0.1% TFA and finally eluted with 1000 µL 0.1% TFA, 80% ACN. The eluate was divided into four aliquots, dried by vacuum centrifugation and stored at -20 °C.

### Off-line peptide fractionation

Offline high-pH High-performance liquid chromatography (HPLC) fractionation was carried out using an UltiMate™ 3000 Nano LC system. One aliquot of each dried TMT set was dissolved in 22 µL of 2.5 mM NH_4_OH, and 20 µL were injected onto an XBridge BEH C_18_ column (130 Å pore size, 4.6 mm inner diameter). Peptide separation occurred over a 65-min gradient at a flow rate of 100 µL/min, with Buffer B (B: 84% ACN, A: H_2_O) increasing from 1 to 45%, and Buffer C (C: 25 mM NH_4_OH) being maintained at 10%. Fractions were collected at 1-min intervals and combined into 24 fractions by alternating across two rows of a 96-well microtiter plate. The column was cleaned with 90% B, 10% C for 10 min, then equilibrated with 1% B, 10% C for another 10 min. The collected eluates were then lyophilized using a vacuum centrifuge and stored at -20 °C.

### Liquid chromatography-mass spectrometry (LC–MS)

Fractionated TMT sets were analyzed on a nano-LC (Ultimate RSLC Nano, Thermo Scientific) equipped with a C_18_ trap column (PepMap Acclaim 300 µm mm * 5 mm, Thermo Scientific) and C_18_ separation column (PepMap Acclaim 75 µm * 500 mm, Thermo Scientific), connected to an Orbitrap Fusion™ Tribrid™ mass spectrometer (Thermo Scientific). Peptides were loaded (loading buffer: 0.05% TFA, 0.1% bovine serum albumin) and separated in accordance with the following gradient: 5 min, 5% B; 8 min, 10% B; 20 min, 25%; 40 min, 35% B; 54 min, 55% B; 56 min, 100% B (Buffer A: 0.1% FA; Buffer B: 84% ACN, 0.1% FA). In positive ion mode, full Orbitrap MS scans were acquired cycling through three mass ranges with a cycle time of 1–2 s (m/z = 375–675, R = 60 k; m/z = 675–975, R = 120 k; m/z = 975–1500, R = 120 k) utilizing the following global parameters: AGC target = 100%, max injection time = 50 ms. Data dependent Orbitrap MS/MS scans for each cycle were recorded with the settings: isolation window = 1.5 m/z, activation type = HCD, R = 50 k, AGC target = 300%, max. injection time = 90 ms.

### Data processing and normalization

Proteome Discoverer Version 2.5.0.400 (Thermo Scientific) was utilized for processing all RAW files. Peak integration for reporter ion quantification was carried out using the most confident centroid integration method with an integration tolerance of 20 ppm. Tryptic and semi-tryptic peptides were identified by searching against the UniProtKB Swiss-Prot (TaxID = 9606, Homo sapiens) database using the SequestHT search engine, with the following search parameters: precursor Δm tolerance = 5 ppm, fragment Δm tolerance = 0.02 Da, max. missed cleavages = 1, minimum peptide length = 6, and fixed modifications including carbamidomethyl and TMTpro (peptide N-terminus, K residues). Percolator was employed for peptide scoring, filtering peptide spectral matches, and ensuring a false discovery rate (FDR) of less than 1%. Peptides were subsequently assembled into proteins and only unique peptides were considered for quantitation. To normalize the data, individual protein abundances were divided by their set-wise overall median. Each resulting protein ratio was then further divided by the sample median to account for deviations in total protein amount. All data were transformed into log_2_-space. Principal component analysis (PCA) and hierarchical clustering were performed on all sample-wise protein abundances to identify potential batch effects and sample outliers. Datasets of the EMIF and iNPH cohort were processed and normalized as previously described [26, 29].

### Weighted protein co-expression network analysis

Weighted protein co-expression networks were constructed with the R package *WGCNA* [30] including all participants of the TRIAD cohort. Proteins with missing values in more than 50% of participants were excluded, and the optimal soft threshold power was selected (R^2^ ~ 0.9, mean/median connectivity < 100). The *WGCNA::blockwiseModules* function was used to construct a signed network with the settings: soft threshold power = 10, deepSplit = 4, corType = bicor, minModuleSize = 10, mergeCutHeight = 0.2, pamRespectsDendro = TRUE, pamStage = TRUE, maxPOutliers = 0.05, reassignThreshold = 0.05. In summary, a robust correlation metric, bicor, was used to compute correlations between all protein pairs. The correlation matrix was then transformed into an adjacency matrix by raising it to the soft threshold power. This adjacency matrix was converted into a topological overlap matrix (TOM), reflecting the interconnectedness of proteins within the proteome. Hierarchical clustering of proteins based on the topological overlap dissimilarity measure (1-TOM) led to cluster construction via dynamic tree cutting. Twelve clusters were identified, including a gray cluster for unassigned proteins. Cluster Eigenproteins, the first principal component of each cluster, were calculated to serve as representative abundance values. Pearson correlation was used to determine protein cluster membership (kME) for each protein (correlation of protein with respective Eigenprotein value). Finally, cluster Eigenproteins were correlated with clinical and analytical parameters using Spearman rank-order correlation. Bonferroni correction was applied to adjust for multiple testing.

### Gene ontology annotation

Gene ontology (GO) analyses of protein clusters were carried out using g:Profiler [31]. The tool performs statistical overrepresentation analysis, determining GO terms that are overrepresented in a selected list of proteins (proteins within a cluster) compared with the background proteome (all proteins identified in the study). To correct for multiple testing, the Bonferroni method was applied with a threshold of *P* < 0.05. GO results were filtered to minimize redundancy and emphasize driver terms. The most representative term was selected for cluster description.

### Statistical analysis

Statistical analyses were conducted using R version 4.1.2. The Kruskal–Wallis test was used to assess the significance of continuous demographic variables, while the Pearson Chi-Square test was applied to categorical variables. Correlations between continuous variables were determined using Spearman rank-order correlation unless otherwise specified. The *limma* package [32] was employed for proteome-wide differential abundance analyses of PET-stratified groups in the TRIAD cohort. Linear models were fitted with protein abundances as outcome variables (minimum number of observations = 20) and PET groups as predictors, adjusting for age and sex. *P*-values were corrected for multiple testing using the Benjamini–Hochberg procedure or Bonferroni procedure, depending on the desired level of stringency. To test the association of proteins with Aβ and tau PET SUVR in all TRIAD cohort participants, linear models were constructed with protein abundances as outcome variables and Aβ and tau PET SUVR as predictors in the same model, while adjusting for age, sex and clinical diagnosis. LASSO regression analysis was performed using the R package *glmnet* (α = 0.7). Summary statistics were illustrated with boxplots showing the median, 25th and 75th percentiles, and whiskers representing measurements from the 5th to the 95th percentiles of non-outlier samples, with outliers plotted beyond the whiskers. Details on all statistical analyses as well as on how existing data was filtered for each analysis can be found in Supplementary table S1.

## Results

### Study design

TMT mass spectrometry was used for untargeted quantitative proteomic analysis of the CSF from 134 individuals of the cross-sectional TRIAD cohort, with available clinical characterization of the study participants, Aβ and tau PET, as well as fluid biomarker measurements (Table 1 A, Supplementary Table S2).

**Table 1:**
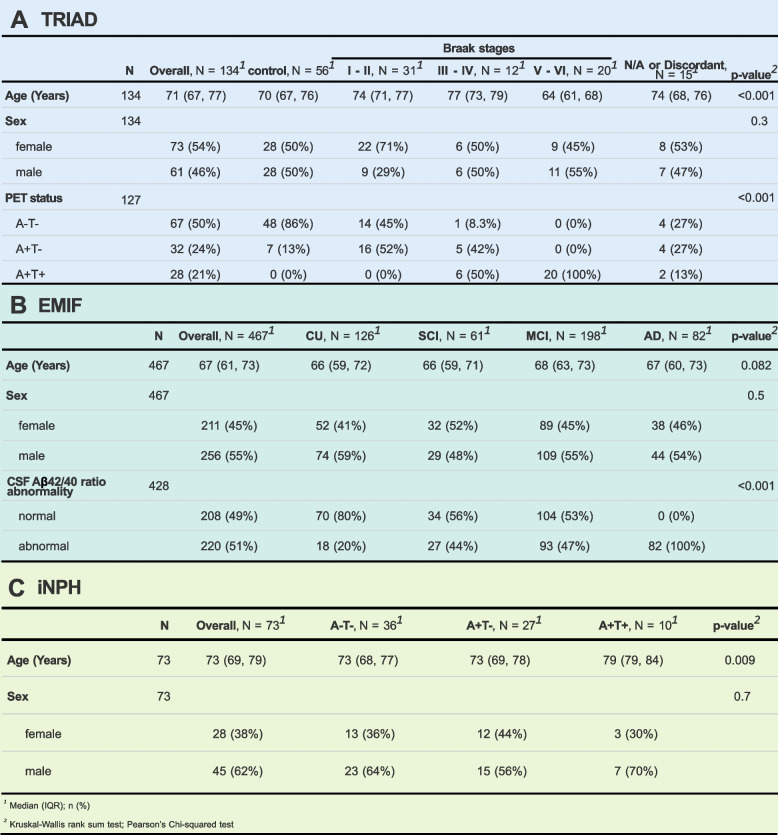
Demographics of the TRIAD, EMIF and iNPH cohorts. For more detailed information of the cohorts refer to the Supplementary material S2-S4. Amyloid and/or tau positivity were evaluated based on different biomarker modalities: PET in the case of TRIAD, CSF fluid biomarker measurements in the case of EMIF and cortical brain biopsy in the case of the iNPH cohort. Abbreviations: TRIAD, Translational Biomarkers in Aging and Dementia; PET, positron emission tomography; EMIF, European Medical Information Framework; iNPH, idiopathic normal pressure hydrocephalus; CU, cognitively unimpaired; SCI, subjective cognitive impairment; MCI, mild cognitive impairment; AD, Alzheimer’s disease

**Fig. 1:**
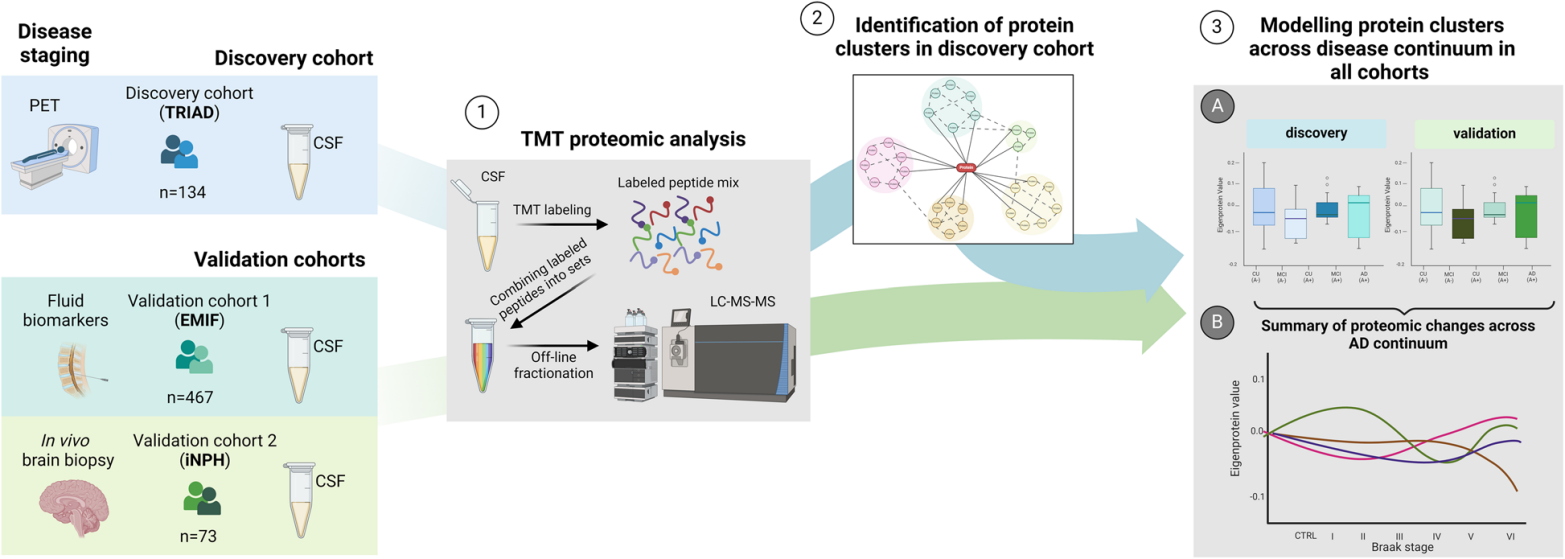
Overview of the study design. CSF samples from the TRIAD cohort (*n* = 134), comprised of PET-staged individuals across the AD continuum, were analyzed with our TMT proteomics workflow (1). Next, clusters of strongly correlating proteins were identified in the TRIAD cohort (2) and plotted across the disease continuum as well as correlated with disease-relevant biomarkers (3). Protein clusters of interest were then validated by evaluating their abundance changes in two additional TMT proteomics validation cohorts, namely EMIF (*n* = 467) and iNPH (*n* = 73) (3A), with study participants placed on the disease continuum via fluid biomarker measurements or in vivo brain biopsy, respectively. Finally, proteomic trends observable in all three cohorts were summarized (3B). Abbreviations: PET, positron emission tomography; TMT, tandem mass tag; LC–MS/MS, liquid chromatography coupled to tandem mass spectrometry; TRIAD, Translational Biomarkers in Aging and Dementia; EMIF, European Medical Information Framework; iNPH, idiopathic normal pressure hydrocephalus

In the TRIAD dataset, which served as discovery cohort, we identified clusters of strongly correlating proteins to reduce the dimensionality of the proteome while maintaining its overall structure (Fig. 1). In a next step, clusters were plotted across the AD continuum and correlated with biomarker measurements of interest to assess their association with different pathologies. Finally, the CSF proteomic datasets of two additional cohorts, previously acquired in-house with the same TMT proteomics workflow, were used to validate relevant findings. Specifically, individuals of the cross-sectional European Medical Information Framework for Alzheimer’s disease (EMIF-AD, henceforth referred to as EMIF) (*n* = 467; Table 1B, Supplementary Table S3) and Kuopio idiopathic normal pressure hydrocephalus (iNPH) (*n* = 73; Table 1C, Supplementary Table S4) cohort were staged utilizing fluid biomarkers and in vivo cortical brain biopsy, respectively, providing orthogonality to the imaging approach in TRIAD. Both cohorts consist of participants along the AD continuum as well as non-AD controls. Notably, all participants in the iNPH cohort have the neurological condition iNPH, which is often comorbid with AD and requires brain surgery, providing a rare opportunity for in vivo brain biopsy for disease staging. By integrating multiple staging paradigms, we aimed to ensure that observed biomarker patterns are not an artifact of a single staging approach. Different staging systems may capture different subgroups of patients; comparisons across cohorts can thus reveal whether biomarker patterns are universal or subtype specific.

### Identification of disease-relevant protein clusters

**Fig. 2:**
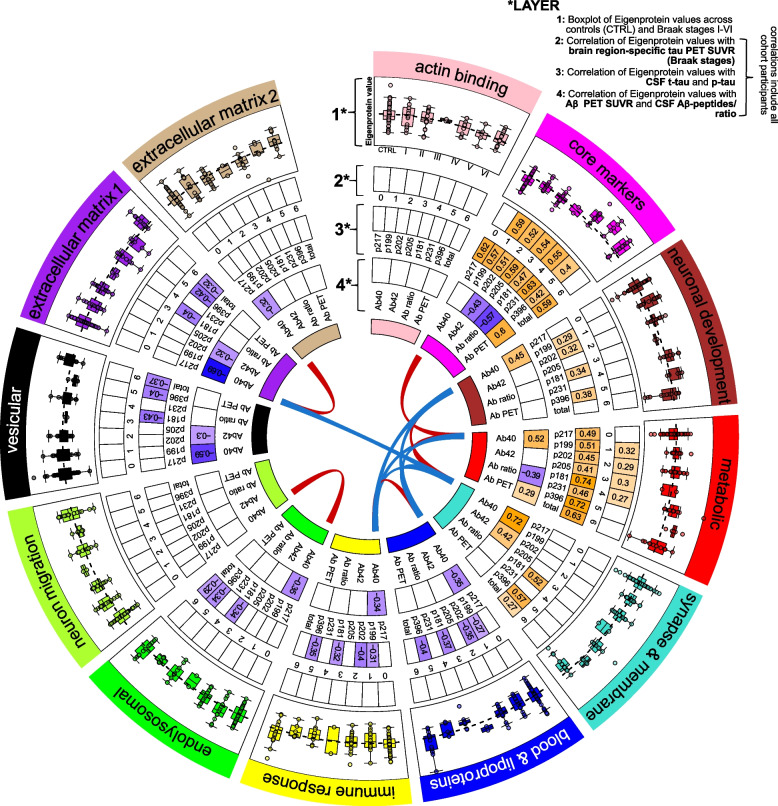
Protein level changes and correlations with biomarker measurements of protein clusters identified in TRIAD. Each segment of the circle represents one of the 12 protein clusters identified in the TRIAD cohort. The outer layer (layer 1) of each segment shows a plot of the cluster’s Eigenprotein values (first principal component of the cluster; z-scored) across tau-negative cognitively unimpaired controls (CTRL) and Braak stages I-VI, with each dot corresponding to an individual. The dashed line denotes the z-scored Eigenprotein value at z-score = 0. All remaining layers [2–4] are dedicated to correlations of the Eigenprotein value with relevant biomarker measurements in all TRIAD study participants. They show the Spearman *ρ* of correlations below the significance threshold *P* < 0.05 after Bonferroni correction. The second layer indicates correlations of the Eigenprotein value with tau PET SUVR in different brain regions, corresponding to the Braak stages I-VI. The third layer shows correlations of the Eigenprotein value with measurements of CSF total-tau, acquired by immunoassay, and tau peptides phosphorylated at different residues, acquired by mass spectrometry. Finally, the inner layer (layer 4) depicts correlations of the Eigenprotein value with CSF Aβ_1-40_, Aβ_1-42_, Aβ ratio and Aβ PET SUVR. Blue and red lines connecting the clusters represent correlations of corresponding Eigenprotein values. The presence of a red line indicates a correlation of *ρ* > 0.6 and *P* < 0.01, while the presence of a blue line shows a correlation of *ρ* < -0.6 and *P* < 0.01. Abbreviations: CTRL, control; PET, positron emission tomography; SUVR, Standardized Uptake Value Ratio

Applying weighted protein co-expression network analysis to the 1506 CSF proteins quantified in > 50% of participants of the TRIAD cohort, we identified 12 clusters containing a median of 36 proteins (Fig. 2, Supplementary Figure S1, Supplementary Table S5). Due to their strong correlation, proteins within the same cluster are likely to reflect similar biological processes in the brain, facilitating a biological interpretation of the clusters. Gene ontology analysis (Supplementary Table S6) showed that constituent proteins mirrored diverse biological functions, ranging from actin binding to synaptic and metabolic processes. Plotting each cluster’s Eigenprotein value, the first principal component of a cluster translating to its representative abundance value, across tau-negative controls and PET Braak-like stages in TRIAD (henceforth simply referred to as Braak stages) revealed a chronology of proteomic changes (Fig. 2). While the ‘endolysosomal’ and ‘neuron migration’ clusters appeared to increase in abundance early on in Braak I, others such as the ‘synapse & membrane’ or ‘extracellular matrix 1’ cluster did not exhibit protein level changes until Braak VI.

To help select clusters with potential disease-relevance, we exploited the rich characterization of study participants in TRIAD and correlated every cluster’s Eigenprotein value with 1. Aβ PET and brain region-specific tau PET SUVR, 2. CSF Aβ_1-40_, Aβ_1-42_, and Aβ ratio, as well as 3. CSF total-tau (t-tau) and tau peptides phosphorylated at diverse amino acid residues (p-tau), across all study participants (Fig. 2). One cluster, termed ‘core markers’ containing known neurodegenerative markers such as YWHAG and UCHL1, showed a strong correlation with t-tau and p-tau species (*ρ* = 0.42–0.63, *P* < 0.0001), Braak I-V SUVR (*ρ* = 0.40–0.59, *P* < 0.0001) and Aβ-ratio (*ρ* = -0.57, *P* < 0.0001), as well as with Aβ PET SUVR (*ρ* = 0.6, *P* < 0.0001). Similarly, the ‘metabolic’ cluster correlated with the Aβ ratio (*ρ* = -0.39, *P* < 0.0001) and all tau species (*ρ* = 0.41–0.74,* P* < 0.0001), the weakest correlation being observed for p-tau_205_, believed to be more reflective of tangle pathology [33]. In line with this, the ‘metabolic’ cluster only exhibited moderately strong correlations with Braak I-IV SUVR (*ρ* = 0.27–0.32, *P* < 0.05).

Other notable clusters included the ‘synapse & membrane’ cluster which correlated with both Aβ_1-40_ (*ρ* = 0.72, *P* < 0.0001), and Aβ_1-42_ (*ρ* = 0.42, *P* < 0.0001) measurements, as well as the ‘immune response’ and ‘blood & lipoproteins’ clusters which showed strong negative correlations with many p-tau epitopes. More detailed correlations including both Aβ-PET-positive and negative individuals (cognitive scores etc.), as well as correlations within Aβ PET-positive individuals only, can be found in Supplementary Figure S2 A-B.

After careful consideration of all correlations and protein level trends across the AD continuum, we chose to further investigate the ‘core markers’, ‘endolysosomal’, ‘metabolic’, ‘synapse & membrane’ and ‘immune response’ clusters. To test if their correlation structure was retained in the EMIF and iNPH cohort, we created correlation matrices of each cluster’s constituent proteins that had a correlation of *ρ* > 0.6 (henceforth referred to as kME) with the cluster’s Eigenprotein value (Supplementary Figure S3 A-J). Indeed, correlations among proteins within a cluster remained strong and therefore proteins with kME > 0.6 were utilized to calculate comparative Eigenprotein values in the EMIF and iNPH cohort.

### Proteins strongly associated with Aβ and tau PET increase early and continue to rise as the disease progresses

**Fig. 3:**
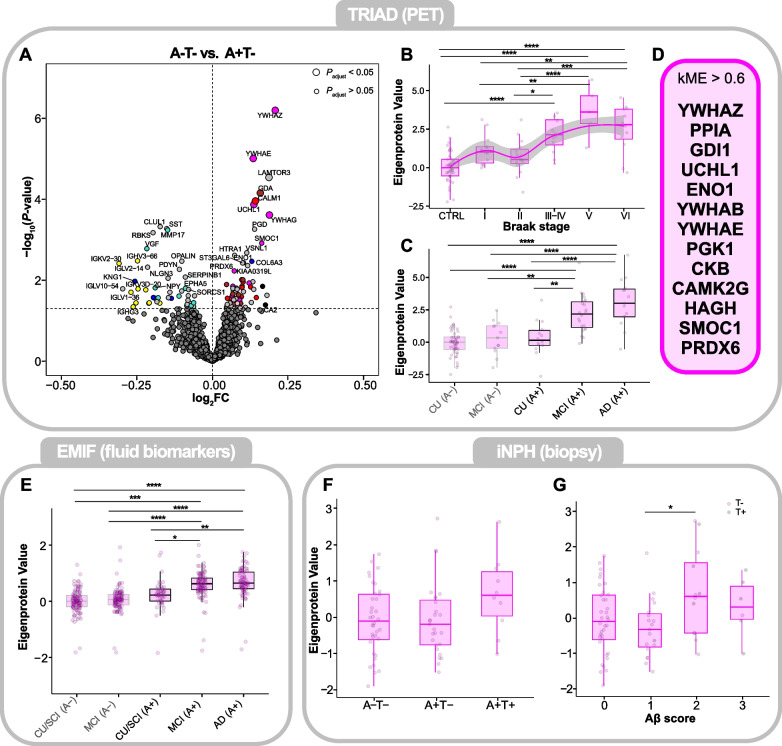
‘Core markers’ protein cluster in the TRIAD, EMIF and iNPH cohorts. **A** Volcano plot showing proteins significantly increased (log_2_FC > 0, *P* < 0.05) and decreased (log_2_FC < 0, *P* < 0.05) in A+T- compared with A-T- individuals in the TRIAD cohort, stratified based on Aβ and tau PET status. Abundance differences were evaluated by fitting linear models, adjusting for age and sex as covariates. Unadjusted *P*-values were plotted. The dot size shows whether the significance threshold of *P* < 0.05 was passed after multiple testing correction by the Benjamini–Hochberg procedure. Proteins are color-coded according to their cluster membership (light gray = no cluster membership) (**B**) Boxplot of ‘Core markers’ Eigenprotein values across controls and Braak stages I-VI in the TRIAD cohort. The protein abundance trend was modelled by a loess-function (shaded area represents the 95% confidence interval). **C** Boxplot of ‘Core markers’ Eigenprotein values across clinical groups, stratified by Aβ PET status, in the TRIAD cohort. **D** Proteins with a correlation coefficient *ρ* > 0.6 with the cluster’s Eigenprotein value, i.e. proteins belonging to the ‘Core markers’ cluster. **E** Boxplot of ‘Core markers’ Eigenprotein values across clinical groups, stratified by CSF Aβ status, in the EMIF cohort. **F** Boxplot of ‘Core markers’ Eigenprotein values across cortical biopsy groups in the iNPH cohort. **G** Boxplot of ‘Core markers’ Eigenprotein values across Aβ-scores, a semiquantitative scale for the Aβ plaque load in the frontal cortex of patients in the iNPH cohort. A gray-colored point indicates the presence of neurofibrillary tangles in the cortical sample of the respective individual. Eigenprotein values of all plots were z-scored to their respective controls. Statistical significances for all boxplots were assessed using linear models, with age and sex as covariates, followed by post-hoc Tukey’s HSD for pairwise comparisons to control the family-wise error rate. *P < 0.05, **P < 0.01, ***P < 0.001, ****P < 0.0001. Abbreviations: CTRL, control; CU/SCI, cognitively unimpaired/subjective cognitive impairment; MCI, mild cognitive impairment; AD, Alzheimer’s disease; A-, Aβ-negative; A + , Aβ-positive

To explore the earliest proteomic changes occurring in AD, we fitted linear models comparing protein levels between A-T- (Aβ-negative, tau-negative) and A+T- (Aβ-positive, tau-negative) individuals, stratified by PET, in the TRIAD cohort. Visualizing the results in a volcano plot and color-coding protein cluster membership (Fig. 3 A), it became apparent that the proteins most strongly increased with Aβ PET positivity (e.g*.*, YWHAZ, YWHAG, UCHL1) belonged to the ‘core markers’ cluster. Examining this cluster more closely, we observed a stepwise increase of the ‘core markers’ Eigenprotein values until Braak stage V (Fig. 3 B) and across the clinical continuum (Fig. 3 C). Abundance differences to controls were significant as early as Braak stage III-IV (*P* < 0.0001), at which point most patients are expected to be Aβ PET-positive [34]. Proteins contained within the ‘core markers’ cluster included many neuronal 14–3-3 proteins (YWHAZ, YWHAB, YWHAG), previously shown to be increased in AD, as well as PPIA, a potential astrocytic marker, and GDI1, a marker of microglial activation, underlining the cell-type diversity of this cluster (Fig. 3 D). Importantly, observed protein level trends in TRIAD could be replicated in the EMIF cohort, where the ‘core markers’ cluster was already elevated at the MCI_A+_ stage compared with CU/SCI_A-_ (cognitively unimpaired/subjective cognitive impairment) individuals (Fig. 3 E). In the iNPH cohort, this trend was less clear, with Eigenprotein values increasing at the A+T+ stage but failing to reach the significance threshold (Fig. 3 F). Looking across the Aβ plaque load in the cortex of iNPH patients, however, a significant increase of the ‘core markers’ proteins could be observed at higher plaque loads (Aβ score 1 vs. 2, *P* < 0.05; Fig. 3 G). Since the weaker trends observed in iNPH were surprising, we investigated CSF Aβ and tau changes across the biopsy stages (Supplementary Figure S4). Indeed, t-tau (Supplementary Figure S4 A, D) and p-tau_181_ (Supplementary Figure S4 B, E) abundances were not elevated until the A+T+ (Aβ-positive, tau-positive) biopsy stage, in line with a previous publication [27], indicating that Aβ pathology detected in possible early-stage AD by sensitive in vivo biopsy may not yet be reflected in the fluid biomarkers. For comparison, changes in available fluid biomarker measurements across the AD continuum in TRIAD and EMIF can be found in Supplementary Figure S4 G-J.

As the Eigenprotein value is only a composite score of all proteins contained within a cluster, we also examined protein level changes of selected proteins (YWHAZ, SMOC1, UCHL1) in TRIAD (Supplementary Figure S5 A-C), EMIF (Supplementary Figure S5 H-J) and iNPH (Supplementary Figure S5 N-P) for validation purposes. Their trends largely reflected the Eigenprotein values, although SMOC1 showed a much stronger increase at the CU/SCI_A+_ stage compared with CU/SCI_A-_ (*P* < 0.0001; Supplementary Figure S5 I) in EMIF.

Lastly, given the strong correlation of the ‘core markers’ cluster with Aβ and tau PET SUVR, we examined the association of proteins with PET by fitting linear models including both Aβ and tau PET SUVR as predictors in the same model (Supplementary Figure S6). Many proteins belonging to the ‘core markers’ cluster showed significant associations with both Aβ and tau PET (YWHAZ, YWHAE, PPIA), while others appeared to be closer linked to Aβ PET only (YWHAB, UCHL1, SMOC1). Abundance plots of these proteins in TRIAD, EMIF and iNPH can be found in Supplementary Figure S5 A-F, H–S while Supplementary Figure S5 G shows the correlation of these proteins with relevant clinical parameters in TRIAD. Especially SMOC1 and YWHAE appeared to be strongly associated with Aβ positivity in the EMIF cohort. Interestingly, we identified one protein, CNN3, not belonging to any cluster but being most strongly associated with Aβ PET (Supplementary Figure S6). Examining CNN3 protein levels in iNPH revealed a strong increase already at the A+T- stage (Supplementary Figure S5 T), though the number of observations was limited and the difference thus non-significant. Unfortunately, CNN3 was not quantifiable in the EMIF cohort, most likely due to its low concentration in CSF.

### Proteins reflecting endo-lysosomal processes increase early and decrease later back to pre-disease levels

**Fig. 4:**
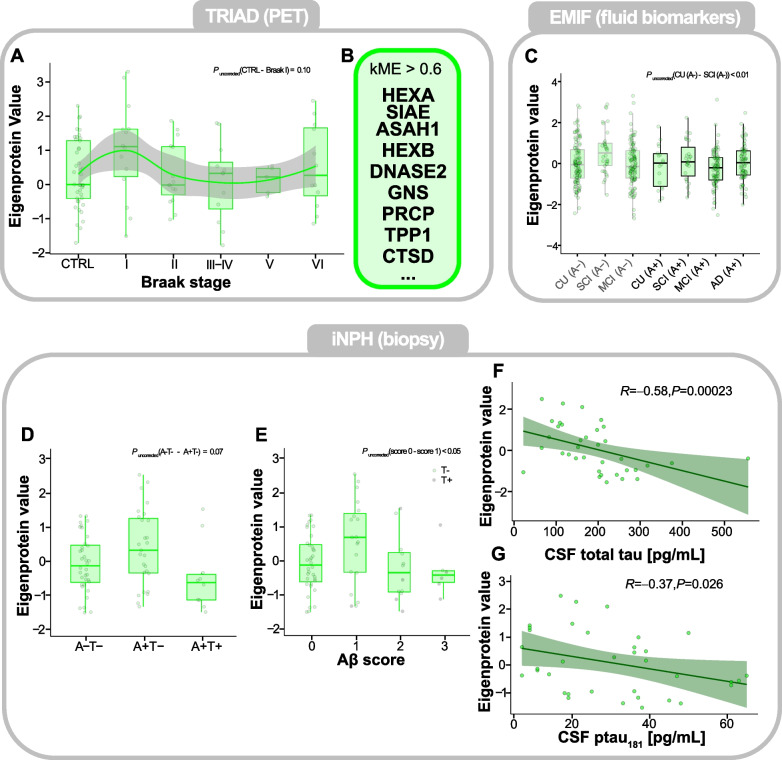
‘Endolysosomal’ protein cluster in the TRIAD, EMIF and iNPH cohorts. **A** Boxplot of ‘Endolysosomal’ Eigenprotein values across controls (CTRL) and Braak stages I-VI in the TRIAD cohort. The protein abundance trend was modelled by a loess-function with the shaded area representing the 95% confidence interval. **B** Proteins with a correlation coefficient *ρ* > 0.6 with the cluster’s Eigenprotein value, i.e. proteins belonging to the ‘Endolysosomal’ cluster. **C** Boxplot of ‘Endolysosomal’ Eigenprotein values across clinical groups, stratified by CSF Aβ status, in the EMIF cohort. **D** Boxplot of ‘Endolysosomal’ Eigenprotein values across cortical biopsy groups in the iNPH cohort. **E** Boxplot of ‘Endolysosomal’ Eigenprotein values across Aβ scores, a semiquantitative scale for the Aβ-plaque load in the frontal cortex of patients in the iNPH cohort. A gray-colored point indicates the presence of neurofibrillary tangles in the cortical sample of the respective individual. **F** Spearman rank-order correlation between the ‘Endolysosomal’ Eigenprotein values and CSF t-tau. **G** Spearman rank-order correlation between the ‘Endolysosomal’ Eigenprotein values and CSF p-tau_181_. Eigenprotein values of all plots were z-scored to their respective controls. Statistical significances for all boxplots were assessed using linear models, with age and sex as covariates, followed by post-hoc Tukey’s HSD for pairwise comparisons to control the family-wise error rate. Unadjusted *P*-values for pairwise comparisons are indicated if relevant. *P < 0.05, **P < 0.01, ***P < 0.001, ****P < 0.0001. Abbreviations: CTRL, control; CU/SCI, cognitively unimpaired/subjective cognitive impairment; MCI, mild cognitive impairment; AD, Alzheimer’s disease; A-, Aβ-negative; A + , Aβ-positive

One protein cluster showing early abundance increases in Braak I (Fig. 4 A) was the ‘endolysosomal’ cluster. It contained proteins, mostly enzymes (Fig. 4 B), known to fulfil different functions in the lysosome, *e.g.*, CTSD, which is regarded as the primary Aβ-degrading enzyme [35]. Although the abundance differences between controls and Braak I were rendered non-significant in TRIAD, a significant increase of the Eigenprotein value could be detected in EMIF in SCI_A-_ individuals compared with MCI_A-_ individuals (*P* < 0.05; Fig. 4 C). When examining the protein levels of HEXB, TPP1 and SIAE, all part of the ‘endolysosomal’ cluster, across all three cohorts (Supplementary Figure S7 A-C, E-M), in EMIF (Supplementary Figure S7 E-G), abundance differences were more pronounced and the mean levels between CU_A-_ and SCI_A-_ as well as between SCI_A-_ and MCI_A-_ differed significantly (*P* < 0.01 -* P* < 0.05) for all proteins. While protein levels were notably higher in SCI_A-_ individuals, they remained unchanged in CU/SCI_A+_ individuals, calling into question whether observed alterations are, in fact, AD-related or rather reflective of other brain-related processes. In the iNPH cohort, the ‘endolysosomal’ cluster showed a marked increase at the A+T- stage compared with A-T- and A+T+ individuals (Fig. 4 D) and was elevated at Aβ score 1 (*P*_*uncorrected*_ < 0.05; Fig. 4 E) before returning to pre-disease levels. Similarly, these trends could be observed for the individual proteins HEXB (Supplementary Figure S7 H,K), TPP1 (Supplementary Figure S7 I,L), and SIAE (Supplementary Figure S7 J,M).

Since we found a significant negative correlation of the ‘endolysosomal’ cluster and constituent proteins with p-tau_181_ (*ρ* =-0.34, *P* < 0.001) and t-tau (*ρ* = -0.29, *P* < 0.01) combining both Aβ-PET-positive and negative individuals in TRIAD (Supplementary Figure S2 B, Figure S7 D), we sought to validate these correlations in iNPH. Indeed, both correlations could be replicated in iNPH (Fig. 4 F, G).

### Levels of immunoglobulins decrease during mid-stages and increase towards the late stages of AD

**Fig. 5:**
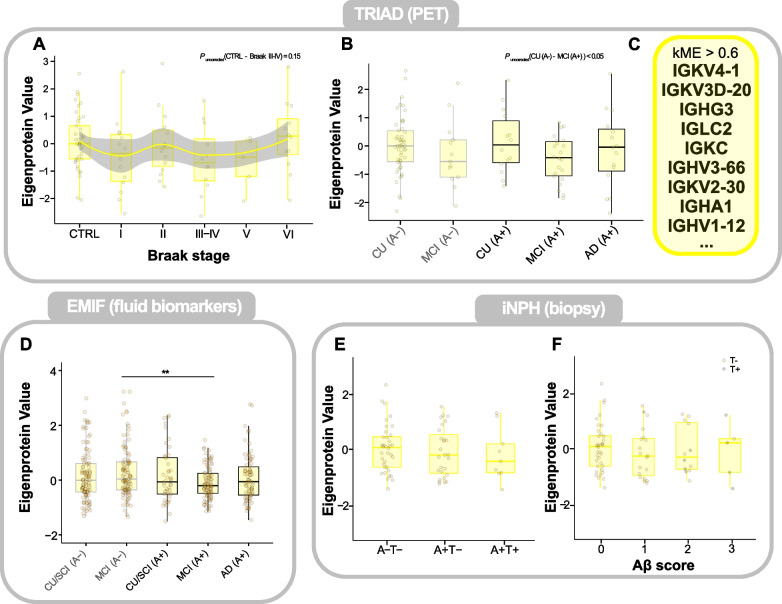
‘Immune response’ protein cluster in the TRIAD, EMIF and iNPH cohorts. **A** Boxplot of ‘Immune response’ Eigenprotein values across controls and Braak stages I-VI in the TRIAD cohort. The protein abundance trend was modelled by a loess-function with the shaded area representing the 95% confidence interval. **B** Boxplot of ‘Immune response’ Eigenprotein values across clinical groups, stratified by Aβ-PET status, in the TRIAD cohort. **C** Proteins with a correlation coefficient *ρ* > 0.6 with the cluster’s Eigenprotein value, i.e. proteins belonging to the ‘Immune response’ cluster. **D** Boxplot of ‘Immune response’ Eigenprotein values across clinical groups, stratified by CSF Aβ status, in the EMIF cohort. **E** Boxplot of ‘Immune response’ Eigenprotein values across cortical biopsy groups in the iNPH cohort. **F** Boxplot of ‘Immune response’ Eigenprotein values across Aβ scores, a semiquantitative scale for the Aβ-plaque load in the frontal cortex of patients in the iNPH cohort. A gray-colored point indicates the presence of neurofibrillary tangles in the cortical sample of the respective individual. Eigenprotein values of all plots were z-scored to their respective controls. Statistical significances for all boxplots were assessed using linear models, with age and sex as covariates, followed by post-hoc Tukey’s HSD for pairwise comparisons to control the family-wise error rate. Unadjusted *P*-values for pairwise comparisons are indicated if relevant. *P < 0.05, **P < 0.01, ***P < 0.001, ****P < 0.0001. Abbreviations: CTRL, control; CU/SCI, cognitively unimpaired/subjective cognitive impairment; MCI, mild cognitive impairment; AD, Alzheimer’s disease; A-, Aβ-negative; A + , Aβ-positive

The proteomic comparison of A-T- with A+T- individuals in the TRIAD cohort (Fig. 3 A) revealed that many proteins decreased in abundance in Aβ PET-positive individuals were immunoglobulins belonging to the ‘immune response’ cluster. When mapped across the Braak stages and clinical groups in TRIAD (Fig. 5 A,B), the ‘immune response’ cluster was decreased in early to mid-stages of AD compared with controls (*P*_*uncorrected*_(CU_A-_—MCI_A+_) < 0.05) and increased back to pre-disease levels in Braak stage VI. Many proteins included in the cluster were immunoglobulin kappa-light and heavy chain variants (Fig. 5 C). Similarly, immunoglobulin levels were decreased in the MCI_A+_ group in EMIF compared with MCI_A-_ (*P* < 0.01; Fig. 5 D). For selected individual proteins (IGKV4-1, IGKV3D-20, IGHG3), this trend was replicable or even stronger (Supplementary Figure S8 A-C, E-M), with a significant difference (*P* < 0.05) being detected comparing IGKV4-1 levels of CU/SCI_A-_ with MCI_A+_ individuals in EMIF (Supplementary Figure S8 E). Notably, all three proteins correlated most strongly with p-tau_202_ in the TRIAD cohort (Supplementary Figure S8 D). In the iNPH cohort, no differences could be observed between either cortical biopsy groups (Fig. 5 E) or across Aβ scores (Fig. 5 F) on the protein cluster level. However, when examining the protein abundances of IGKV4-1, IGKV3D-20, and IGHG3 individually (Supplementary Figure S8 H-M), a trend towards decreased levels in A+T- individuals compared with the A-T- groups emerged, albeit insignificant.

### Mid- to late-stage proteomic changes are linked to metabolic disturbances and synaptic dysfunction

**Fig. 6:**
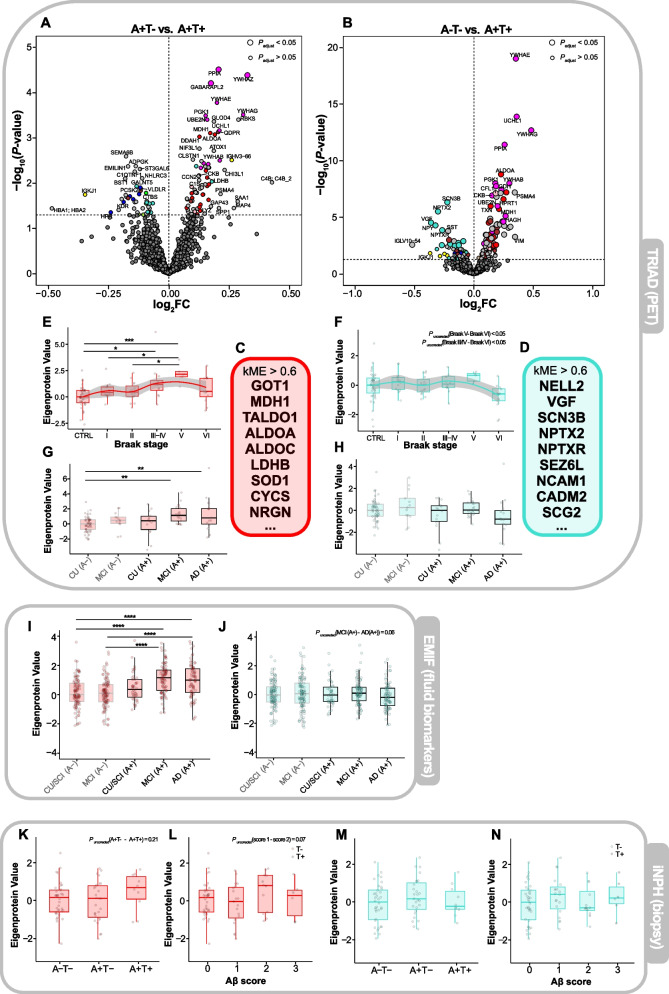
The ‘metabolic’ and ‘synapse & membrane’ protein clusters in the TRIAD, EMIF and iNPH cohorts. Volcano plot highlighting proteins significantly increased (log_2_FC > 0, *P* < 0.05) and decreased (log_2_FC < 0, *P* < 0.05) in A+T+ compared with (**A**) A+T- or (**B**) A-T- individuals in the TRIAD cohort, stratified by Aβ and tau PET status. Linear models, adjusting for age and sex, were used to assess abundance differences, and unadjusted *P*-values were plotted. Dot size indicates whether significance (*P* < 0.05) was maintained after Benjamini–Hochberg multiple testing correction. Proteins are color-coded by cluster membership (light gray = no cluster membership). **C**/**D** Proteins with a correlation coefficient *ρ* > 0.6 with the cluster’s Eigenprotein value, i.e., proteins belonging to the ‘metabolic’ (**C**) or ‘synapse & membrane’ cluster (**D**). **E** Boxplot of ‘metabolic’ or (**F**) ‘synapse & membrane’ Eigenprotein values across controls and Braak stages I-VI in the TRIAD cohort; protein abundance trends were modelled by a loess-function (95% confidence interval shaded). **G** Boxplot of ‘metabolic’ or (**H**) ‘synapse & membrane’ Eigenprotein values across clinical groups, stratified by Aβ PET status, in the TRIAD cohort. **I** Boxplot of ‘metabolic’ or (**J**) ‘synapse & membrane’ Eigenprotein values across clinical groups, stratified by CSF Aβ status, in the EMIF cohort. **K** Boxplot of ‘metabolic’ or (**M**) ‘synapse & membrane’ Eigenprotein values across cortical biopsy groups in the iNPH cohort. **L** Boxplot of ‘metabolic’ or (**N**) ‘synapse & membrane’ Eigenprotein values across Aβ scores. Gray points indicate neurofibrillary tangles in cortical samples. Eigenprotein values of all plots were z-scored to their respective controls. Statistical significances for all boxplots were assessed using linear models, with age and sex as covariates, followed by post-hoc Tukey’s HSD for pairwise comparisons to control the family-wise error rate. Unadjusted *P*-values for pairwise comparisons are indicated if relevant. *P < 0.05, **P < 0.01, ***P < 0.001, ****P < 0.0001. Abbreviations: CTRL, control; CU/SCI, cognitively unimpaired/subjective cognitive impairment; MCI, mild cognitive impairment; AD, Alzheimer’s disease; A-, Aβ-negative; A + , Aβ-positive

Protein clusters, which appeared to change later in the temporal progression of AD, were the ‘metabolic’ and ‘synapse & membrane’ clusters. Volcano plots comparing the A+T- and A-T- groups with A+T+ individuals, respectively (Fig. 6 A, B), revealed that many upregulated proteins mapped to the ‘metabolic’ cluster (Fig. 6 C) and, conversely, many downregulated proteins belonged to the ‘synapse & membrane’ cluster (Fig. 6 D). The Eigenprotein values of both clusters showed a strong positive correlation with each other (*ρ* = 0.76, *P* < 0.001; Supplementary Figure S9), indicating that they likely follow similar trajectories. Mapping their temporal profiles across Braak stages (Fig. 6 E,F) and clinical groups (Fig. 6 G,H) showed that both clusters displayed a sharp decrease in Braak stage VI. While the ‘metabolic cluster’ levels increased linearly up to Braak stage V (control – Braak V, *P* < 0.01), the ‘synapse & membrane’ cluster remained largely unchanged throughout stages I-V.

Examining both clusters in the EMIF cohort (Fig. 6 I,J) confirmed these trajectories, with ‘metabolic’ proteins being especially elevated in MCI_A+_ individuals (CU_A-_ – MCI_A+_, *P* < 0.0001) before dropping slightly in AD_A+_, and ‘synapse & membrane’ protein levels being slightly, but non-significantly decreased in AD_A+_ compared with the other groups. These trends were further mirrored in the individual ‘metabolic’ proteins ALDOA, MDH1, and GOT1 (Supplementary Figure S10 A-M). Proteins contained within the ‘synapse & membrane’ cluster (e.g*.*, VGF, NPTX2, SCN3B; Supplementary Figure S11 A-M) overall showed similar trends towards decrease in late-stage AD, though individual abundance changes were more pronounced, reaching clear significance in the CU/SCI_A-_ vs. AD_A+_ comparison.

In the iNPH cohort, the temporal profiles were less clear (Fig. 6 K-N), however, a trend towards increase in mid-stages and a consecutive drop in later stages was observable for the ‘metabolic’ cluster (Fig. 6 L) and selected constituent proteins (Supplementary Figure S10 H-M). Looking at individual proteins of the ‘synapse & membrane’ cluster, VGF and SCN3B showed a significant decrease already in earlier stages comparing A-T- with A+T- individuals (*P* < 0.05, Supplementary Figure S11 H-M).

### Compilation of results for sequential CSF proteomic changes observed across three AD cohorts

**Fig. 7:**
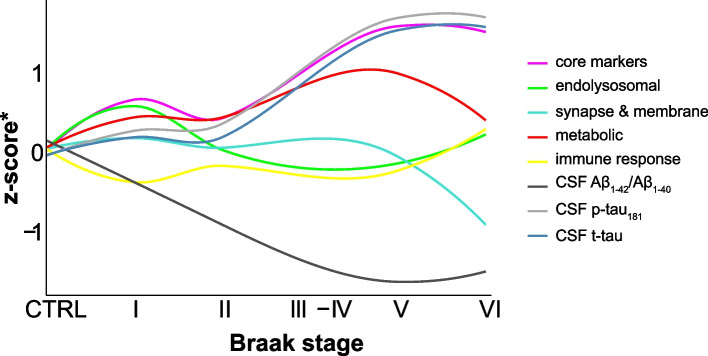
Temporal profiles of protein clusters across Braak stages. Summary plot of loess-functions of protein clusters ‘core markers’ (magenta), ‘endolysosomal’ (green), ‘immune response’ (yellow), ‘metabolic’ (red), and ‘synapse & membrane’ (turquoise), as well as CSF Aβ-ratio (black), p-tau_181_ (gray) and t-tau (steelblue) across controls (CTRL) and Braak stages in TRIAD. *The y-axis represents z-scored Eigenprotein values in the case of protein clusters and compressed z-scored abundances in the case of the core CSF biomarkers. Trajectories were additionally shifted in y-direction for better comparability. Thus, displayed fold-changes or baseline values are not comparable among protein clusters and core biomarkers

A summary of all temporal profiles discussed in this study, including the CSF core biomarkers Aβ ratio, p-tau_181_ and t-tau, can be viewed in Fig. 7. The plot emphasizes the dynamic nature of proteomic changes across Braak stages, showing their temporal fluctuations. The greatest protein level alterations could be observed for the ‘metabolic’, ‘synapse & membrane’ as well as ‘core markers’ cluster, which closely followed the trajectories of p-tau_181_ and t-tau. The ‘endolysosomal’ and ‘immune response’ clusters, on the other hand, displayed more subtle changes. Although the profiles of each cluster and biomarker differed, there appeared to be two common inflection points at Braak stages II and V, where abundance changes began to be more pronounced or the directionality of change switched, respectively. Generally, protein abundances appeared to plateau or even drop at Braak stage VI. Of note, conclusions regarding Braak V should be drawn with caution as the number of observations for this stage was limited (*n* = 5). Similarly, only two individuals were classified as Braak stage III; therefore, Braak stages III and IV were combined for visualization and modeling purposes.

To evaluate the predictive utility of specific proteins for staging along the AD continuum, we conducted LASSO regression analysis in the larger EMIF cohort (Supplementary Figure S12 A–B, D–E) and validated the results in the independent TRIAD cohort (Supplementary Figure S12 C, F). As anticipated, several proteins associated with the 'core markers', 'metabolic', and 'synapse & membrane' clusters were selected for distinguishing between preclinical and prodromal AD (e.g., YWHAB, GDI1, SMOC1, UCHL1, NPTX2, DDAH1, ENO2; Supplementary Figure S12 A) and between prodromal AD and AD dementia (e.g., VGF, NPTXR, UCHL1, PCSK1, SPON1; Supplementary Figure S12 D). ROC analysis demonstrated that the resulting protein panels outperformed conventional clinical parameters (age and *APOE* status) as well as the CSF core biomarkers, achieving AUC values ranging from 0.69 to 0.86 (Supplementary Figure S12 B–C, E–F).

## Discussion

In this study, we used TMT proteomics to map the abundance profile of CSF proteins across the AD continuum in three independent cohorts. We identified clusters of correlated proteins that reflected different pathophysiological aspects of the disease and determined their association with the fluid AD core biomarkers as well as Aβ and tau pathologies through PET imaging and cortical biopsies. Our study revealed that many CSF proteins undergo dynamic and non-linear changes as Aβ and tau pathologies progress.

One of the earliest detectable changes involved an abundance increase in proteins belonging to the ‘core markers’ cluster, which strongly correlated with fluid and imaging biomarkers. The ‘core markers’ cluster showed a stepwise increase across the AD continuum in all three cohorts and was also the cluster with the highest fold change between AD patients and controls. It contained a heterogeneous group of proteins, likely derived from different cell types and reflecting general neurodegenerative processes e.g., synapse-enriched 14–3-3 isoforms (ζ, β, ε) [36] and CAMK2G [37], possibly mirroring synaptic dysfunction; neuronal UCHL1 implicated in proteasomal protein degradation [38, 39]; microglia-enriched GDI1 reflecting neuroinflammatory responses [40]; and putative astrocyte and pericyte-enriched PPIA/CypA [41], involved in blood–brain barrier (BBB) integrity loss [42]. Many of the proteins included in the ‘core markers’ cluster have been previously reported to be increased in the CSF of AD patients [43–45], but also in other neurodegenerative diseases such as Lewy body diseases (LBD) [46, 47] and frontotemporal dementia (FTD) [40, 48]. Although likely non-specific to AD, the 14–3-3 proteins and PPIA demonstrated a strong association with both Aβ and tau PET. While this could be the result of a generally high correlation between neurodegenerative processes and neuropathology, 14–3-3-proteins have been shown to directly interact with AD pathology by co-localizing with NFTs [49]. A recent proteomics study additionally demonstrated a strong relation of a 14–3-3 protein, YWHAQ, to tau PET load [18]. Interestingly, the ‘core markers’ cluster was the only cluster to strongly correlate with p-tau_205_, a p-tau species that was recently shown to be reflective of NFT pathology [33]. Further, among others, the proteins UCHL1, SMOC1 and CNN3 were significantly associated with Aβ PET and Aβ status in our validation cohorts. Indeed, studies demonstrated that UCHL1 regulates BACE1 and APP degradation, leading to decreased Aβ production [50–52]. In accordance with our findings, SMOC1 has been recently shown to predict longitudinal Aβ PET status conversion [53], was associated with Aβ PET and Aβ-positivity in several proteomics studies [17, 18] and was identified as Aβ plaque component [54]. SMOC1 is mainly expressed in oligodendrocytes [55] and their precursor cells in the human brain, which resonates with the recently reported role of oligodendrocytes in Aβ plaque formation [56]. Finally, CSF levels of CNN3 in AD have not yet been examined, however, a recent study implicated the *CNN3* gene in the regulation of microglia-mediated Aβ phagocytosis [57].

The group of proteins associated with endo-lysosomal processes increased in the early stages of AD and decreased back to pre-disease levels at the later stages. Importantly, endo-lysosomal dysfunction has long been recognized as a key feature of AD, with endocytic pathway perturbations even preceding Aβ deposition [58, 59]. Studies have reported that various acid hydrolases (CTSD, CTSB, HEXA), which help reduce Aβ accumulation [35, 60] and are part of our 'endolysosomal cluster’, are present in extracellular Aβ plaques [61, 62]. This suggests that lysosomal content is liberated from cells and might therefore be increased in the CSF of early-stage AD patients. Indeed, one study measuring endo-lysosomal proteins in the CSF of controls and AD patients noted a weak trend towards increased abundances in prodromal AD [63]. Another recent proteomics study described an elevation of endo-lysosomal proteins at earlier AD stages, followed by a decrease [64]; in line with our observations. Strikingly, in the EMIF cohort, we observed elevated endo-lysosomal protein levels in the SCI_A-_ group only, while abundances in the CU/SCI_A+_ groups remained unchanged. A recent meta-analysis highlighted that SCI patients with Aβ abnormality were at increased risk of progressing to AD, while SCI in the absence of Aβ pathology might be indicative of other causes [65]. Although the conditions causing SCI can be manifold [66], a speculation reconciling our results observed across three cohorts could be primary age-related tauopathy (PART), characterized by limited tau accumulation with no or sparse neuritic plaques [67]. It could be hypothesized that a rise in endo-lysosomal proteins reflects an early compensation mechanism preceding tau accumulation, however, more studies are needed to determine the relationship between increased lysosomal protein levels and age-related processes.

One surprising finding of this study was that a fraction of the detected immunoglobulins in CSF, part of the ‘immune response’ cluster and likely derived from plasma, were reduced at the MCI_A+_ stage, both in the TRIAD and EMIF cohort. Conversely, increased levels of immunoglobulins have been shown in the CSF of AD patients [68], possibly as a result of BBB damage [69]. Further investigations would be needed to explain these discrepancies but the negative correlation of immunoglobulins with several p-tau species suggest a link to AD pathophysiology.

Proteins that increased at the MCI_A+_ stage and then slightly decreased/ plateaued at the AD stage mapped to the ‘metabolic’ cluster and included sugar metabolism-related proteins such as GOT1, MDH1 and TALDO1 as well as superoxide dismutases SOD1 and SOD2. This result was in accordance with previous proteomics studies [14, 15, 44, 45], and confirms that sugar metabolism appears to play a role in the pathogenesis of AD [70], particularly considering that diabetes mellitus type 2 is a known risk factor for AD [71]. Finally, we identified a protein cluster, predominantly comprised of synaptic (*e.g*., NPTX2, SV2B, CHGA) [72–74] and membrane-associated proteins, that appeared to decrease at the later stages of AD. Reduced levels of synaptic proteins in AD, but also in LBD and FTD, have been reported in many studies to date [46, 75] and are a common feature of neurodegenerative diseases, reflective of progressive synaptic loss. Of note, both the ‘metabolic’ and ‘synapse & membrane’ cluster were strongly associated with CSF p-tau and Aβ measurements, further emphasizing that their abundance alterations parallel relevant pathological processes.

The highlight of this study includes the profiling of proteomic changes across Braak stages, affording a higher degree of granularity compared to similar CSF proteomics studies [14, 64]. As a result, we found that CSF protein levels are subject to considerable fluctuations over the disease course, sharing certain inflection points which could potentially represent different phenotypical presentations across the disease continuum. Most notably, protein levels appeared to plateau or even sharply decrease at Braak VI. This has previously been reported for different p-tau species [34] and might be a feature of terminal-stage AD. In summary, we could replicate our results in EMIF and, to a lesser degree, in the iNPH cohort. Different groups of proteins were changed at distinct stages which points towards a potential use of these proteins in disease staging. This was further highlighted by our LASSO regression analysis, which suggested that, among others, the proteins UCHL1, YWHAB, NPTX2 and NPTXR might have utility in disease staging or in the context of clinical trials, as they likely reflect different biological processes.

Although TMT mass spectrometry is a powerful tool for the proteomic profiling of clinical cohorts, as evidenced by this and other studies [15, 17], it is important to acknowledge that batch effects can potentially confound the results. To address this, we applied careful normalization procedures to minimize such effects as much as possible. Another limitation of our study is that, although the iNPH cohort offers the rare opportunity of in vivo cortical biopsy staging, the presence of another neurological disorder influencing CSF dynamics [76] might have adverse effects on protein levels. Neuropathological examination is more sensitive than either fluid or imaging measurements, which may explain partially discordant results and cautions sweeping comparisons across the three cohorts. Nonetheless, the iNPH cohort represents the only source of direct histopathological confirmation of amyloid and tau pathology, positioning it as a critical validation reference for biomarker-based staging. In TRIAD, sample sizes for individual Braak stages were relatively low (Braak III, *n* = 2; Braak V, *n* = 5), which must be taken into consideration when assessing minute changes in protein levels. Finally, while the protein cluster approach facilitates a simplified interpretation of the proteome, it comes at the cost of decreased sensitivity. This is evident when comparing individual protein trends with that of the cluster’s Eigenprotein value, both with respect to abundance fold changes and correlation strength. In addition, the Eigenprotein value of large clusters, like the ‘synapse & membrane’ cluster, might not adequately represent abundances of all constituent proteins. Nevertheless, our study was able to identify robust proteomic changes replicable in several cohorts, mitigating small sample sizes and other drawbacks discussed above.

## Conclusion

In summary, our study 1. confirms and suggests novel biomarker candidates associated with Aβ and tau PET, 2. shows the temporal profile of different groups of CSF proteins across the AD continuum in three independent cohorts and 3. highlights the dynamic nature of proteomic changes. The latter bears important implications for the design of biomarker discovery studies and the interpretation of biomarkers in the context of the AD continuum. Proteomic profiles could also serve to further characterize patients in screening for clinical trials or to evaluate biological changes in response to different drug targets. Finally, our findings suggest the utility of different protein groups in disease staging, reflecting pathophysiological processes that could in the future replace our ‘X’.

## Supplementary Information

Supplementary Material 1.

Supplementary Material 2.

Supplementary Material 3.

Supplementary Material 4.

## Data Availability

Raw mass spectrometry results of the TRIAD proteomics dataset have been deposited to the ProteomeXchange Consortium via the PRIDE partner repository with the dataset identifier PXD062585 and 10.6019/PXD062585. Similarly, raw mass spectrometry results of the EMIF-AD proteomics dataset can be accessed on ProteomeXchange (dataset identifiers PXD019910 and 10.6019/PXD019910). Raw data for the iNPH dataset can be obtained upon reasonable request to the Broad Institute (Broad Data Use Oversight System). Normalized abundances with associated clinical data can be requested by researchers from the respective cohort owners: TRIAD (https://triad.tnl-mcgill.com/contact-us/), EMIF-AD MBD (https://emif-catalogue.eu/), and iNPH (https://duos.broadinstitute.org/dataset/DUOS-000144).

## Abbreviations

AD: Alzheimer’s disease
Aβ: Amyloid
ACN: Acetonitrile
CDR: Clinical Dementia Rating
CSF: Cerebrospinal fluid
CU: Cognitively unimpaired
DOC: Sodium deoxycholate
EMIF-AD: European Medical Framework for Alzheimer’s Disease
GO: Gene ontology
HCl: Hydrochloric acid
HPLC: High-performance liquid chromatography
iNPH: Idiopathic normal pressure hydrocephalus
LC-MS: Liquid chromatography-mass spectrometry
MCI: Mild cognitive impairment
NFTs: Neurofibrillary tangles
PET: Positron emission tomography
p-tau: Phosphorylated tau
SCI: Subjective cognitive impairment
SPE: Solid phase extraction
SUVR: Standardized Uptake Value Ratio
TCEP: Tris(2-carboxyethyl)phosphine
TFA: Trifluoroacetic acid
TMT: Tandem mass tag
TRIAD: Translational Biomarkers in Aging and Dementia
