# Supplementary material for: Cerebrospinal fluid proteome profiling across the Alzheimer’s disease continuum: a step towards solving the equation for ‘X’
Source: Mol Neurodegener. 2025 May 6;20:52. doi: 10.1186/s13024-025-00841-0 (PMC12057231; doi:10.1186/s13024-025-00841-0)
Supplement: Supplementary file 2 — Supplementary Material 2. [file 13024_2025_841_MOESM2_ESM.pdf]

Supplementary data file

Inventory

Weiner *et al*. Cerebrospinal fluid proteome profiling across the Alzheimer's disease continuum: A step towards solving the equation for 'X'.

Table S2. Detailed demographics of the TRIAD cohort.

Table S3. Detailed demographics of the EMIF cohort.

Table S4. Detailed demographics of the iNPH cohort.

Figure S1. Cluster dendrogram of protein clusters identified in TRIAD.

Figure S2. Detailed correlation heatmap of analytical and clinical parameters with protein clusters in (A) Aβ-PET positive patients only and (B) all participants in the TRIAD cohort.

Figure S3. Correlation matrices of each cluster's constituent proteins with kME > 0.6 (as identified in TRIAD) in the iNPH (A, C, E, G, I) and EMIF (B, D, F, H, J) cohorts.

Figure S4. Concordance of cortical biopsy stages with CSF core biomarkers t-tau (A, D), p-tau181 (B, E) and β-Amyloid1-42 (C, F) in the iNPH cohort, as well as CSF core biomarker changes across the AD continuum in TRIAD (G-I) and EMIF (J).

Figure S5. Boxplots of six constituent proteins of the 'core markers' cluster, YWHAZ (A, H, N), SMOC1 (B, I, O), UCHL1 (C, J, P), PPIA (D, K, Q), YWHAE (E, L, R), YWHAB (F, M, S) and CNN3 (T, no cluster) across the AD continuum in the TRIAD (A-F), EMIF (H-M) and iNPH cohort (N-T).

Figure S6. Association of proteins with Aβ and tau PET SUVR.

Figure S7. Boxplots of three constituent proteins of the 'endolysosomal' cluster, HEXB (A, E, H, K), TPP1 (B, F, I, L), and SIAE (C, G, J, M) across the AD continuum in the TRIAD (A-C), EMIF (E-G) and iNPH cohort (H-M).

Figure S8. Boxplots of three constituent proteins of the 'immune response' cluster, IGKV4-1 (A, E, H, K), IGKV3D-20 (B, F, I, L), and IGHG3 (C, G, J, M) across the AD continuum in the TRIAD (A-C), EMIF (E-G) and iNPH cohort (H-M).

Figure S9. Correlation matrix of cluster Eigenprotein values.

Figure S10. Boxplots of three constituent proteins of the 'metabolic' cluster, ALDOA (A, E, H, K), MDH1 (B, F, I, L), and GOT1 (C, G, J, M) across the AD continuum in the TRIAD (A-C), EMIF (E-G) and iNPH cohort (H-M).

Figure S11. Boxplots of three constituent proteins of the 'synapse & membrane' cluster, VGF(A, E, H, K), NPTX2 (B, F, I, L), and SCN3B (C, G, J, M) across the AD continuum in the TRIAD (A-C), EMIF (E-G) and iNPH cohort (H-M).

Figure S12. Selection of individual proteins using LASSO regression to distinguish between stages of Alzheimer's disease (AD): preclinical vs. prodromal AD (A–C), and prodromal AD vs. AD dementia (D–F).

Table S2. Detailed demographics of the TRIAD cohort. Abbreviations: TRIAD, Translational Biomarkers in Aging and Dementia; CU, cognitively unimpaired; MCI, mild cognitive impairment; AD, Alzheimer's disease; PET, positron emission tomography; SUVR, standardized uptake value; ROI, region of interest; MMSE, mini-mental state examination.

				Braak stages				
	**N**	**Overall**, N = 134[1]	**control**, N = 56[1]	**I - II**, N = 31[1]	**III - IV**, N = 12[1]	**V - VI**, N = 20[1]	**N/A or Discordant**, N = 15[1]	**p-value**[2]
**Age (Years)**	134	71 (67, 77)	70 (67, 76)	74 (71, 77)	77 (73, 79)	64 (61, 68)	74 (68, 76)	<0.001
**Sex**	134							0.3
female		73 (54%)	28 (50%)	22 (71%)	6 (50%)	9 (45%)	8 (53%)	
male		61 (46%)	28 (50%)	9 (29%)	6 (50%)	11 (55%)	7 (47%)	
**clinical diagnosis**	134							<0.001
CU		75 (56%)	42 (75%)	21 (68%)	1 (8.3%)	1 (5.0%)	10 (67%)	
MCI		39 (29%)	14 (25%)	8 (26%)	9 (75%)	5 (25%)	3 (20%)	
AD		20 (15%)	0 (0%)	2 (6.5%)	2 (17%)	14 (70%)	2 (13%)	
**PET status**	127							<0.001
A-T-		67 (50%)	48 (86%)	14 (45%)	1 (8.3%)	0 (0%)	4 (27%)	
A+T-		32 (24%)	7 (13%)	16 (52%)	5 (42%)	0 (0%)	4 (27%)	
A+T+		28 (21%)	0 (0%)	0 (0%)	6 (50%)	20 (100%)	2 (13%)	
**Aβ PET SUVR**	128	1.45 (1.29, 2.28)	1.30 (1.23, 1.41)	1.72 (1.37, 2.27)	2.39 (1.85, 2.51)	2.42 (2.21, 2.59)	2.22 (1.37, 2.65)	<0.001
**Tau PET meta-ROI SUVR**	128	0.91 (0.83, 1.09)	0.82 (0.76, 0.88)	0.94 (0.87, 0.98)	1.24 (1.18, 1.29)	2.63 (2.25, 2.96)	1.02 (0.93, 1.08)	<0.001
**CSF Aβ42/40 ratio**	134	0.064 (0.044, 0.093)	0.091 (0.077, 0.097)	0.062 (0.048, 0.086)	0.044 (0.037, 0.052)	0.041 (0.034, 0.048)	0.048 (0.041, 0.080)	<0.001
**CSF p-tau$_{181}$**	134	47 (34, 67)	34 (28, 43)	49 (39, 62)	75 (62, 101)	98 (76, 123)	57 (44, 63)	<0.001
**CSF t-tau**	134	351 (273, 471)	285 (223, 341)	353 (271, 424)	459 (404, 633)	679 (476, 801)	395 (319, 473)	<0.001
**MMSE**	133	29 (26, 30)	29 (28, 30)	29 (28, 30)	27 (24, 29)	24 (19, 28)	28 (22, 29)	<0.001
***APOE* ε4 status**	131							<0.001
none		78 (60%)	42 (76%)	19 (61%)	4 (33%)	6 (32%)	7 (50%)	
heterozygous		47 (36%)	13 (24%)	10 (32%)	7 (58%)	10 (53%)	7 (50%)	
homozygous		6 (4.6%)	0 (0%)	2 (6.5%)	1 (8.3%)	3 (16%)	0 (0%)	
**ethnicity**	92							
white (not hispanic or latino)		92 (100%)	38 (100%)	21 (100%)	7 (100%)	18 (100%)	8 (100%)	

[1] Median (IQR); n (%)

[2] Kruskal-Wallis rank sum test; Pearson's Chi-squared test

Table S3. Detailed demographics of the EMIF cohort. Abbreviations: EMIF, European Medical Information Framework; MMSE, mini-mental state examination; CU, cognitively unimpaired; SCI, subjective cognitive impairment; MCI, mild cognitive impairment; AD, Alzheimer's disease.

	N	**Overall**, N = 467[1]	**CU**, N = 126[1]	**SCI**, N = 61[1]	**MCI**, N = 198[1]	**AD**, N = 82[1]	p-value[2]
**Age (Years)**	467	67 (61, 73)	66 (59, 72)	66 (59, 71)	68 (63, 73)	67 (60, 73)	0.082
**Sex**	467						0.5
0		211 (45%)	52 (41%)	32 (52%)	89 (45%)	38 (46%)	
1		256 (55%)	74 (59%)	29 (48%)	109 (55%)	44 (54%)	
**CSF Aβ42/40 ratio abnormality**	428						<0.001
normal		208 (49%)	70 (80%)	34 (56%)	104 (53%)	0 (0%)	
abnormal		220 (51%)	18 (20%)	27 (44%)	93 (47%)	82 (100%)	
**CSF Aβ42/40 ratio**	428	0.060 (0.045, 0.094)	0.087 (0.071, 0.096)	0.070 (0.047, 0.095)	0.072 (0.045, 0.097)	0.045 (0.040, 0.051)	<0.001
**MMSE**	466	28.0 (25.0, 29.0)	29.0 (28.0, 30.0)	29.0 (27.0, 29.0)	27.0 (25.3, 29.0)	23.0 (19.3, 25.0)	<0.001
***APOE* ε4 status**	407						0.002
none		199 (49%)	61 (59%)	35 (57%)	77 (48%)	26 (32%)	
at least one allele		208 (51%)	43 (41%)	26 (43%)	84 (52%)	55 (68%)	

[1] Median (IQR); n (%)

[2] Kruskal-Wallis rank sum test; Pearson's Chi-squared test

Table S4. Detailed demographics of the iNPH cohort. Abbreviations: iNPH, idiopathic normal pressure hydrocephalus; MMSE, mini-mental state examination.

	N	**Overall**, N = 73[1]	**A-T-**, N = 36[1]	**A+T-**, N = 27[1]	**A+T+**, N = 10[1]	**p-value**[2]
**Age (Years)**	73	73 (69, 79)	73 (68, 77)	73 (69, 78)	79 (79, 84)	0.009
**Sex**	73					0.7
F		28 (38%)	13 (36%)	12 (44%)	3 (30%)	
M		45 (62%)	23 (64%)	15 (56%)	7 (70%)	
**Aβ score**	73					<0.001
0		36 (49%)	36 (100%)	0 (0%)	0 (0%)	
1		19 (26%)	0 (0%)	18 (67%)	1 (10%)	
2		12 (16%)	0 (0%)	8 (30%)	4 (40%)	
3		6 (8.2%)	0 (0%)	1 (3.7%)	5 (50%)	
**Tau score**	73					<0.001
0		63 (86%)	36 (100%)	27 (100%)	0 (0%)	
1		8 (11%)	0 (0%)	0 (0%)	8 (80%)	
2		1 (1.4%)	0 (0%)	0 (0%)	1 (10%)	
3		1 (1.4%)	0 (0%)	0 (0%)	1 (10%)	
**CSF $A\beta_{1\text{-}42}$**	72	671 (562, 852)	788 (673, 914)	567 (425, 666)	622 (459, 774)	<0.001
**CSF p-tau_{181}**	71	26 (14, 38)	25 (13, 36)	23 (12, 36)	43 (37, 61)	0.005
**CSF t-tau**	71	170 (128, 217)	148 (128, 209)	155 (110, 203)	232 (208, 311)	<0.001
**comorbidities**	73					0.3
Vascular Cognitive Impairment		4 (5.5%)	2 (5.6%)	1 (3.7%)	1 (10%)	
**Charlson age comorbidity index**	73					0.2
1		4 (5.5%)	4 (11%)	0 (0%)	0 (0%)	
2		17 (23%)	8 (22%)	8 (30%)	1 (10%)	
3		40 (55%)	20 (56%)	15 (56%)	5 (50%)	
4		12 (16%)	4 (11%)	4 (15%)	4 (40%)	
**MMSE**	72	22.0 (20.0, 26.0)	24.0 (21.0, 26.0)	20.0 (17.5, 26.0)	20.0 (19.0, 23.3)	0.045
***APOE* status**	73					0.001
ε2/ε3		10 (14%)	8 (22%)	1 (3.7%)	1 (10%)	
ε2/ε4		3 (4.1%)	0 (0%)	3 (11%)	0 (0%)	
ε3/ε3		44 (60%)	26 (72%)	14 (52%)	4 (40%)	
ε3/ε4		13 (18%)	2 (5.6%)	8 (30%)	3 (30%)	
ε4/ε4		3 (4.1%)	0 (0%)	1 (3.7%)	2 (20%)	

[1] Median (IQR); n (%)

[2] Kruskal-Wallis rank sum test; Fisher's exact test

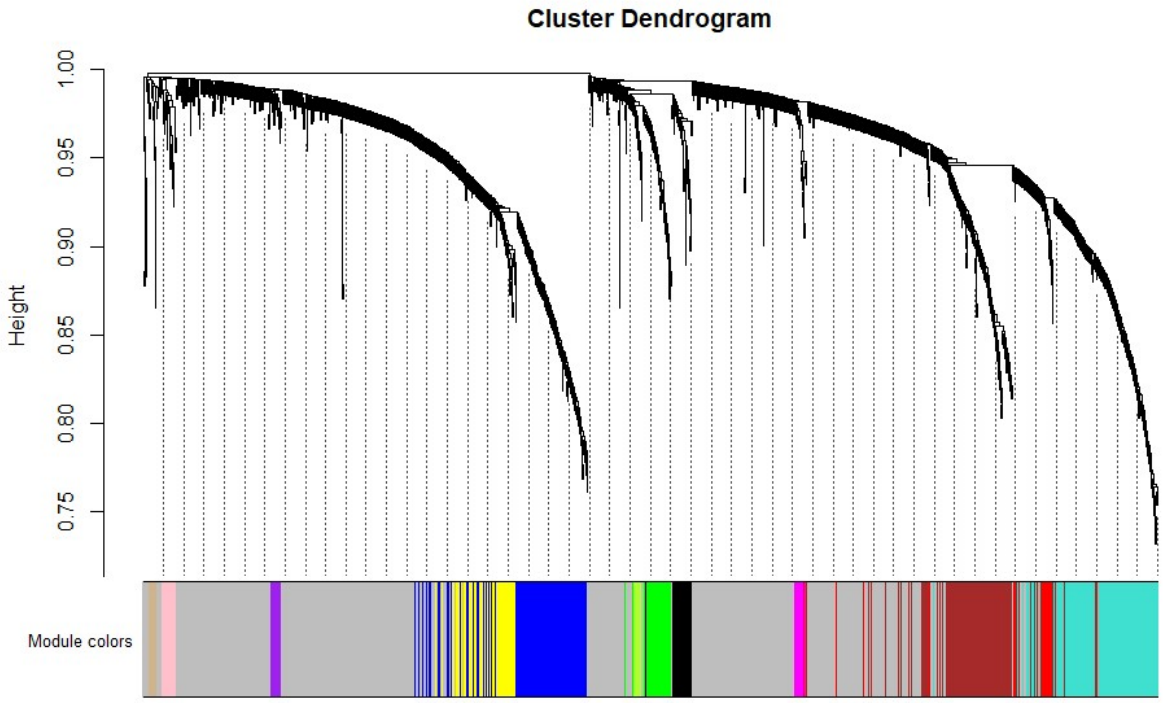

Figure S1. Cluster dendrogram of protein clusters identified in TRIAD. Cluster dendrogram of the 12 color-coded protein clusters identified by average linkage hierarchical clustering of proteins based on their topological overlap measures. Pink, 'actin binding'; magenta, 'core markers'; brown, 'neuronal development'; red, 'metabolic'; turquoise, 'synapse & membrane'; blue, 'blood & lipoproteins'; yellow, 'immune response'; green, 'endolysosomal'; greenyellow, 'neuron migration'; black, 'vesicular'; purple, 'extracellular matrix 1'; tan, 'extracellular matrix 2'.

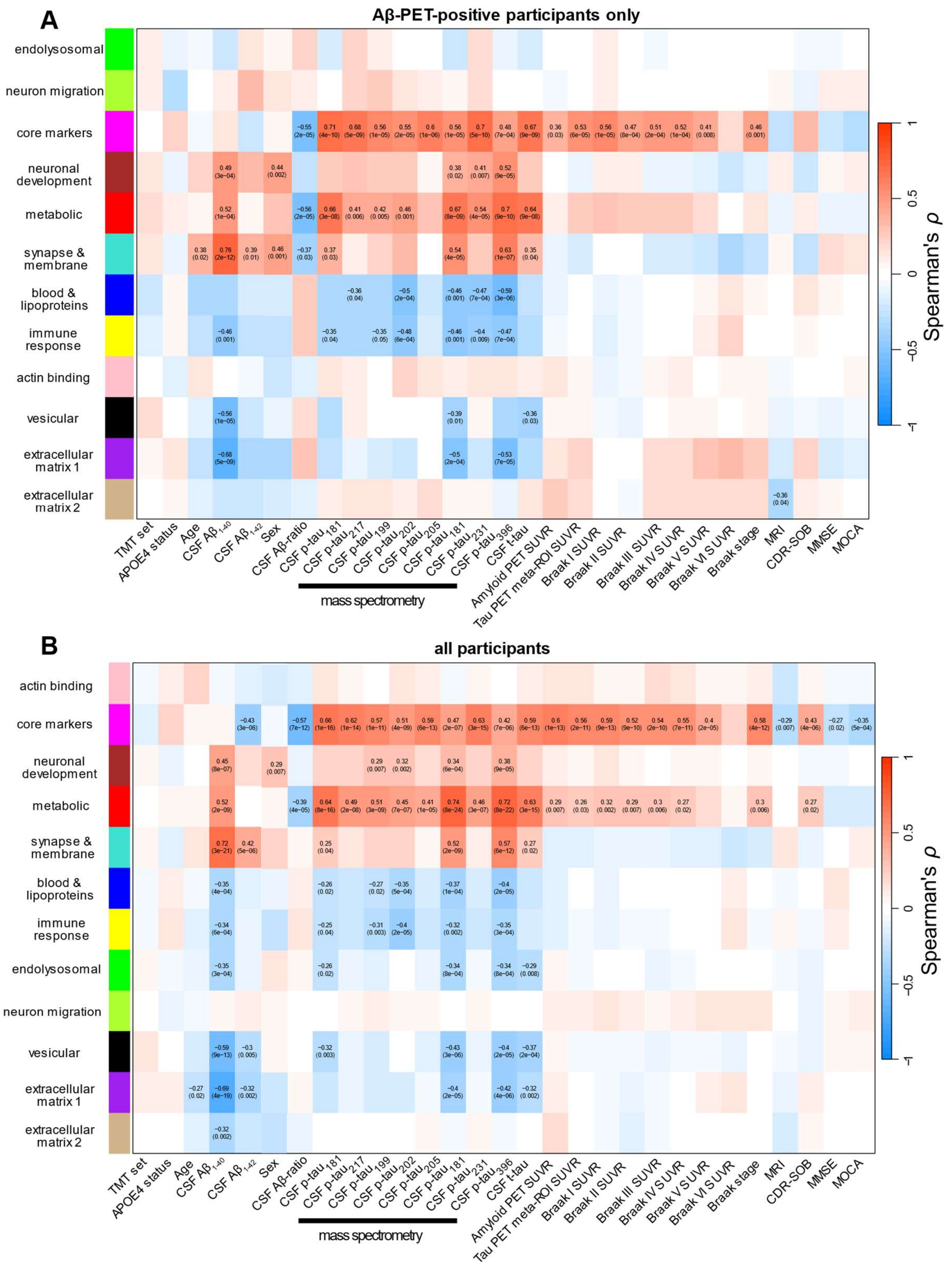

Figure S2. Detailed correlation heatmap of analytical and clinical parameters with protein clusters in (A) Aβ-PET-positive patients only and (B) all participants in the TRIAD cohort. Spearman's rank-order correlation was performed with consecutive Bonferroni correction. Only significant correlations ($P<0.05$) are displayed. The upper value in each box corresponds to the Spearman ρ while the value in parentheses shows the corrected P-value. All fluid biomarker measures were obtained via immunoassay unless otherwise indicated. Abbreviations: PET, positron emission tomography; ROI, region of interest; SUVR, standardized uptake value ratio; MRI, magnetic resonance imaging; CDR-SOB, clinical dementia rating-sum of boxes; MMSE, mini-mental state examination; MOCA, Montreal Cognitive Assessment.

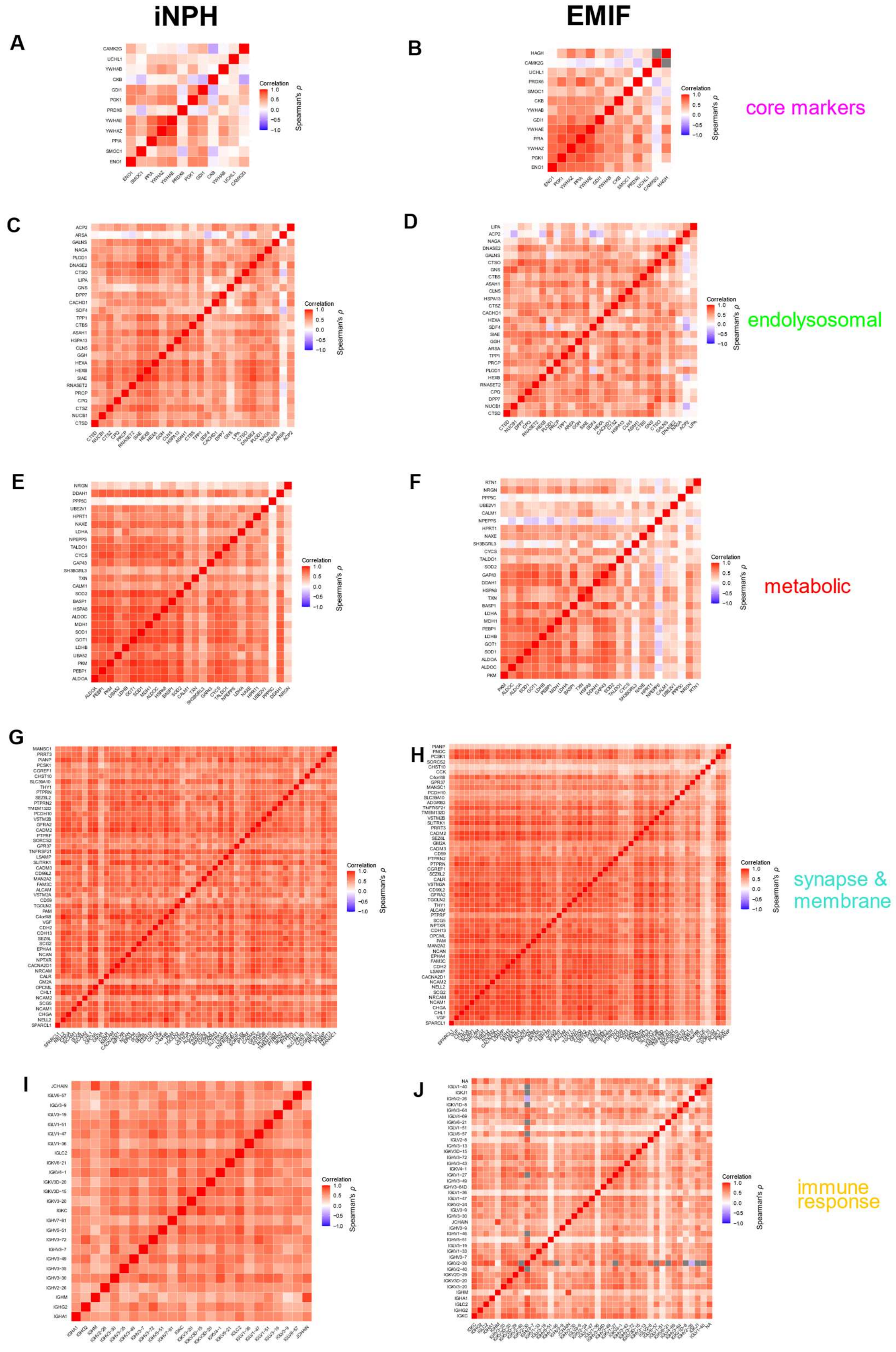

Figure S3. Correlation matrices of each cluster's constituent proteins with kME > 0.6 (as identified in TRIAD) in the iNPH (A, C, E, G, I) and EMIF (B, D, F, H, J) cohorts. Spearman rank-order correlation was performed and the obtained Spearman's ρ was color-coded. Proteins are indicated using their accession number. Correlation of proteins belonging to the 'core markers' (A-B), 'endolysosomal' (C-D), 'metabolic' (E-F), 'synaptic & membrane' (G-H) and 'immune response' (I-J) cluster.

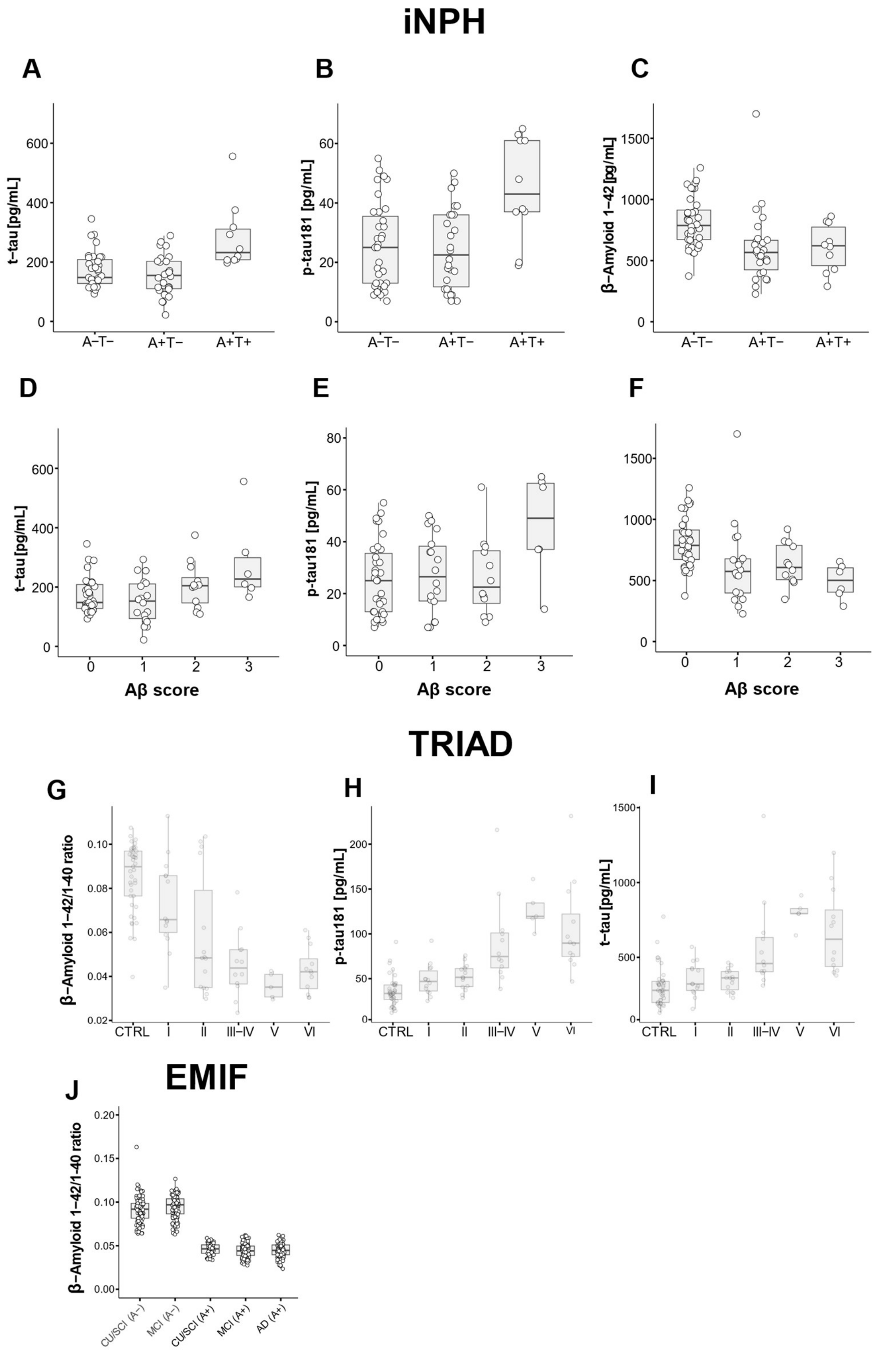

Figure S4. Concordance of cortical biopsy stages with CSF core biomarkers t-tau (A, D), p-tau$_{181}$ (B, E) and β-Amyloid$_{1-42}$ (C, F) in the iNPH cohort, as well as CSF core biomarker changes across the AD continuum in TRIAD (G-I) and EMIF (J). (A-F) CSF core biomarker levels were plotted across cortical Aβ /tau positivity stages (A-C) or across Aβ scores (D-F) in the iNPH cohort, indicating respective plaque load in the frontal cortex. (G-I) CSF core biomarker levels were plotted across Braak-like stages, as evaluated by tau PET in the TRIAD cohort. (J) The CSF Aβ ratio was plotted across clinical groups in the EMIF cohort. Note that the Aβ status was evaluated in accordance with the Aβ ratio, hence, a perfect separation can be observed. Significances are not indicated.

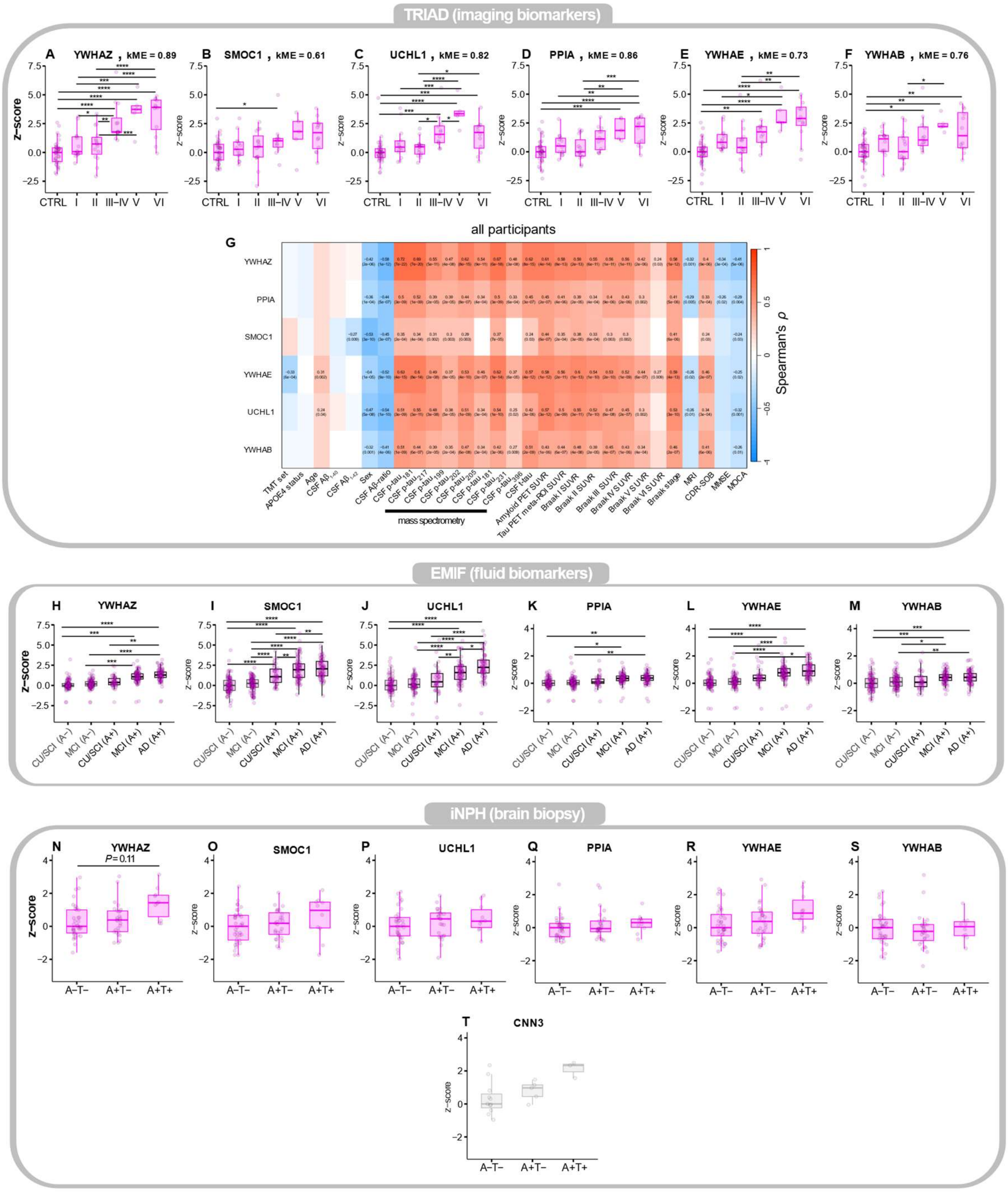

Figure S5. Boxplots of six constituent proteins of the 'core markers' cluster, YWHAZ (A, H, N), SMOC1 (B, I, O), UCHL1 (C, J, P), PPIA (D, K, Q), YWHAE (E, L, R), YWHAB (F, M, S) and CNN3 (T, no cluster) across the AD continuum in the TRIAD (A-F), EMIF (H-M) and iNPH cohort (N-T). (G) Correlation heatmap of relevant parameters of the TRIAD cohort with six constituent proteins of the 'core markers' cluster. (A-F, H-T) Z-scored protein abundances (z-scored to respective reference group) were plotted across clinical groups, stratified by tau PET in the TRIAD cohort (A-F), CSF Aβ status in the EMIF cohort (H-M), and across cortical biopsy groups (N-T) in iNPH. Statistical significances for all boxplots were assessed using linear models, with age and sex as covariates, followed by post-hoc Tukey's HSD for pairwise comparisons to control the family-wise error rate. *P<0.05, **P<0.01, ***P<0.001, ****P<0.0001. Abbreviations: CU/SCI, cognitively unimpaired/subjective cognitive impairment; MCI, mild cognitive impairment; AD, Alzheimer's disease; A-, Aβ-negative; A+, Aβ-positive.

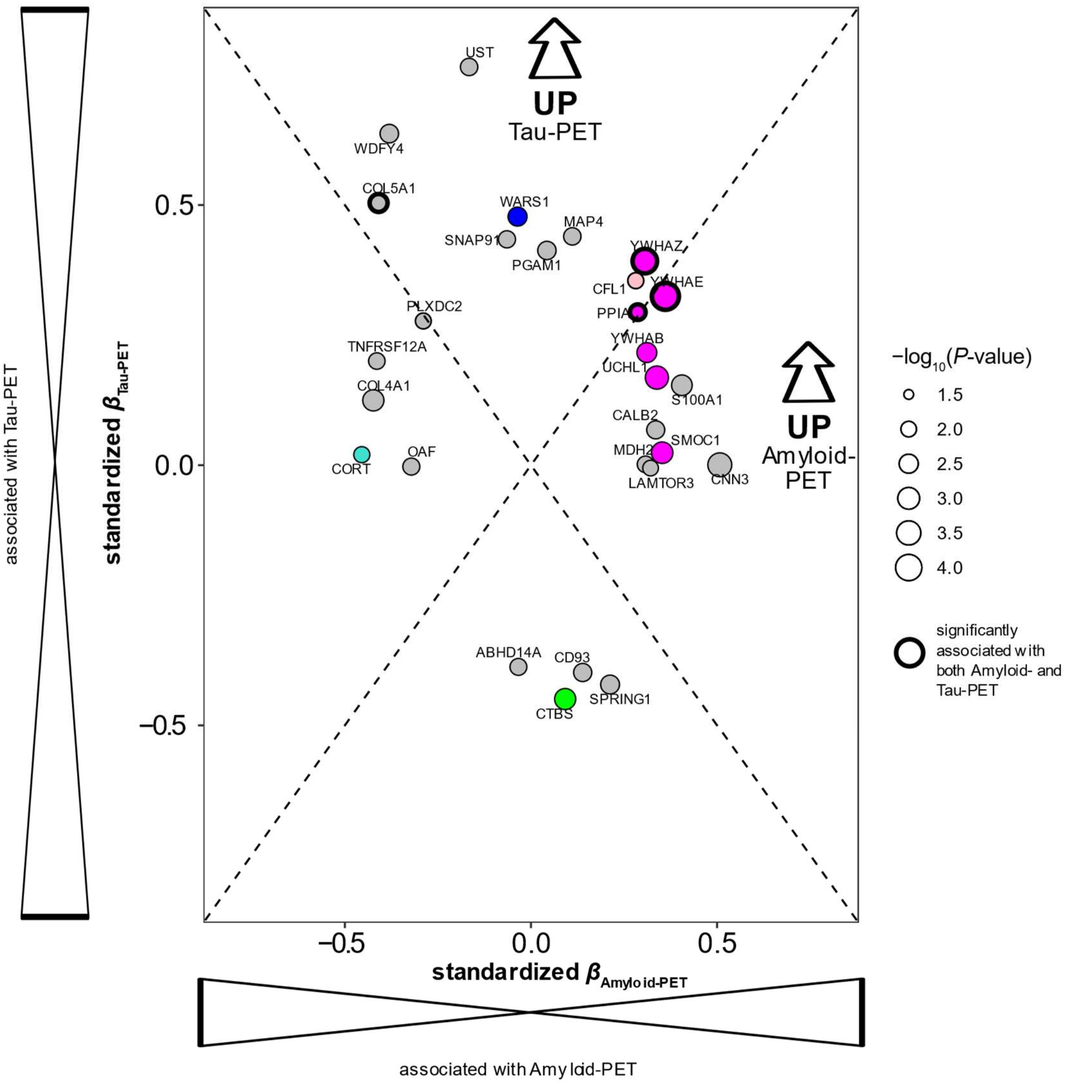

Figure S6. Association of proteins with Aβ and tau PET SUVR. Linear models were fitted with protein abundances as the outcome variable and Aβ and tau PET SUVR as predictors, including age, sex, and clinical diagnosis as covariates. Significant associations with either Aβ or tau PET, or both (bold circle), were identified at an uncorrected $P<0.01$. These associations are shown by plotting the standardized regression coefficients of Aβ PET SUVR (x-axis) against those of tau PET SUVR (y-axis). Proteins varying along the x-axis are strongly associated with Aβ PET, while proteins varying along the y-axis have a stronger association with tau PET. Proteins located along the dotted line are equally associated with both Aβ and tau PET. The size of the circle corresponds to the respective significance level. Proteins were color-coded according to the protein cluster they belong to: magenta, ‘core markers’; pink, ‘actin binding’; blue, ‘blood & lipoproteins’; turquoise, ‘synapse & membrane’; green, ‘endolysosomal’; gray, no cluster assigned. Abbreviations: PET, positron emission tomography; SUVR, standardized uptake value ratio.

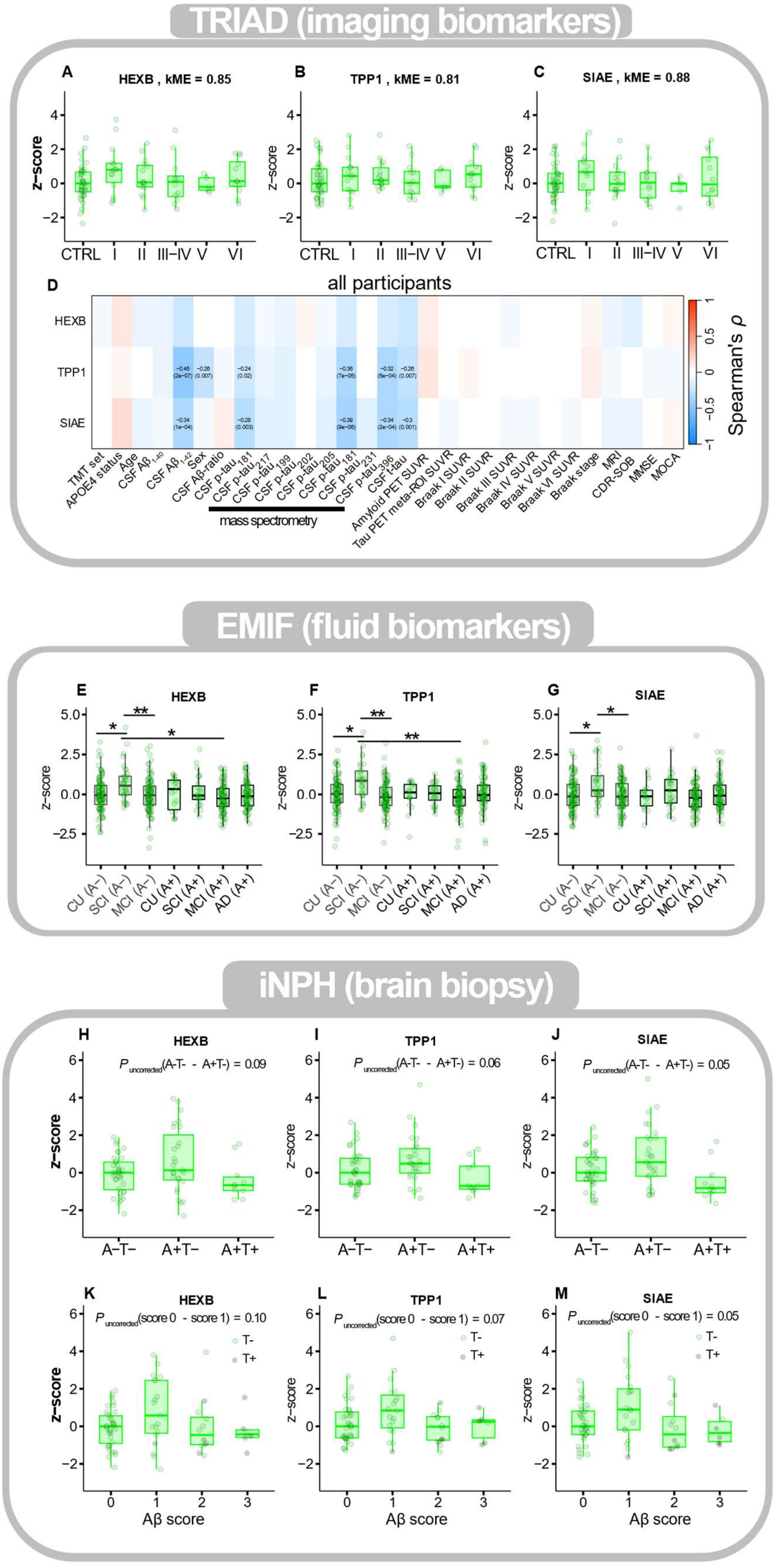

Figure S7. Boxplots of three constituent proteins of the 'endolysosomal' cluster, HEXB (A, E, H, K), TPP1 (B, F, I, L), and SIAE (C, G, J, M) across the AD continuum in the TRIAD (A-C), EMIF (E-G) and iNPH

cohort (H-M). (D) Correlation heatmap of relevant parameters of the TRIAD cohort with three constituent proteins of the 'endolysosomal' cluster. (A-C, E-M) Z-scored protein abundances (z-scored to respective reference group) were plotted across clinical groups, stratified by tau PET in the TRIAD cohort (A-C), CSF Aβ status in the EMIF cohort (E-G), and across cortical biopsy groups (H-J) or Aβ scores (K-M), a semiquantitative scale for the Aβ-plaque load in the frontal cortex of patients, in the iNPH cohort. A gray-colored point indicates the presence of neurofibrillary tangles in the cortical sample of the respective individual. Statistical significances for all boxplots were assessed using linear models, with age and sex as covariates, followed by post-hoc Tukey's HSD for pairwise comparisons to control the family-wise error rate. Unadjusted *P*-values for pairwise comparisons are indicated if relevant. **P*<0.05, ***P*<0.01, ****P*<0.001, *****P*<0.0001. Abbreviations: CU/SCI, cognitively unimpaired/subjective cognitive impairment; MCI, mild cognitive impairment; AD, Alzheimer's disease; A-, Aβ-negative; A+, Aβ-positive.

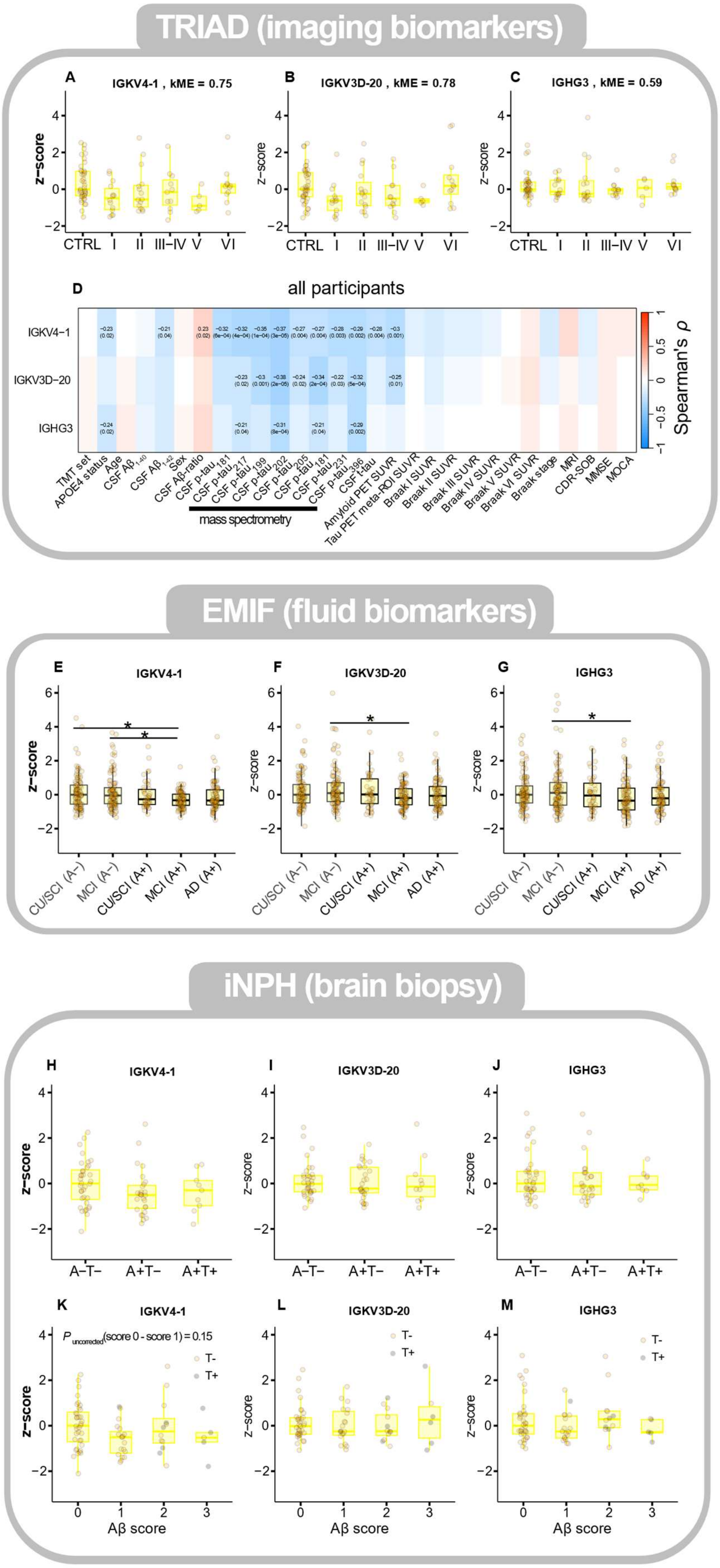

TRIAD (imaging biomarkers)
A
IGKV4-1 , kME = 0.75
B
IGKV3D-20 , kME = 0.78
C
IGHG3 , kME = 0.59
z−score
CTRL I II III−IV V VI
D
all participants
IGKV4−1
IGKV3D−20
IGHG3
Spearman's ρ
TMT set
APOE4 status
Age
CSF Aβ1-40
CSF Aβ1-42
Sex
CSF Aβ-ratio
CSF p-tau181
CSF p-tau217
CSF p-tau199
CSF p-tau202
CSF p-tau205
CSF p-tau181
CSF p-tau231
CSF p-tau396
CSF t-tau
Amyloid PET SUVR
Tau PET meta-ROI SUVR
Braak I SUVR
Braak II SUVR
Braak III SUVR
Braak IV SUVR
Braak V SUVR
Braak VI SUVR
Braak stage
MRI
CDR-SOB
MMSE
MOCA
mass spectrometry
EMIF (fluid biomarkers)
E
IGKV4-1
F
IGKV3D-20
G
IGHG3
z−score
CU/SCI (A−)
MCI (A−)
CU/SCI (A+)
MCI (A+)
AD (A+)
iNPH (brain biopsy)
H
IGKV4-1
I
IGKV3D-20
J
IGHG3
z−score
A−T−
A+T−
A+T+
K
IGKV4-1
P uncorrected (score 0 - score 1) = 0.15
T-
T+
L
IGKV3D-20
M
IGHG3
Aβ score

Figure S8. Boxplots of three constituent proteins of the 'immune response' cluster, IGKV4-1 (A, E, H, K), IGKV3D-20 (B, F, I, L), and IGHG3 (C, G, J, M) across the AD continuum in the TRIAD (A-C), EMIF (E-G) and iNPH cohort (H-M). (D) Correlation heatmap of relevant parameters of the TRIAD cohort with three constituent proteins of the 'immune response' cluster. (A-C, E-M) Z-scored protein abundances (z-scored to respective reference group) were plotted across clinical groups, stratified by tau PET in the TRIAD cohort (A-C), CSF Aβ status in the EMIF cohort (E-G), and across cortical biopsy groups (H-J) or Aβ scores (K-M), a semiquantitative scale for the Aβ-plaque load in the frontal cortex of patients, in the iNPH cohort. A gray-colored point indicates the presence of neurofibrillary tangles in the cortical sample of the respective individual. Statistical significances for all boxplots were assessed using linear models, with age and sex as covariates, followed by post-hoc Tukey's HSD for pairwise comparisons to control the family-wise error rate. Unadjusted *P*-values for pairwise comparisons are indicated if relevant. *P<0.05, **P<0.01, ***P<0.001, ****P<0.0001. Abbreviations: CU/SCI, cognitively unimpaired/subjective cognitive impairment; MCI, mild cognitive impairment; AD, Alzheimer's disease; A-, Aβ-negative; A+, Aβ-positive.

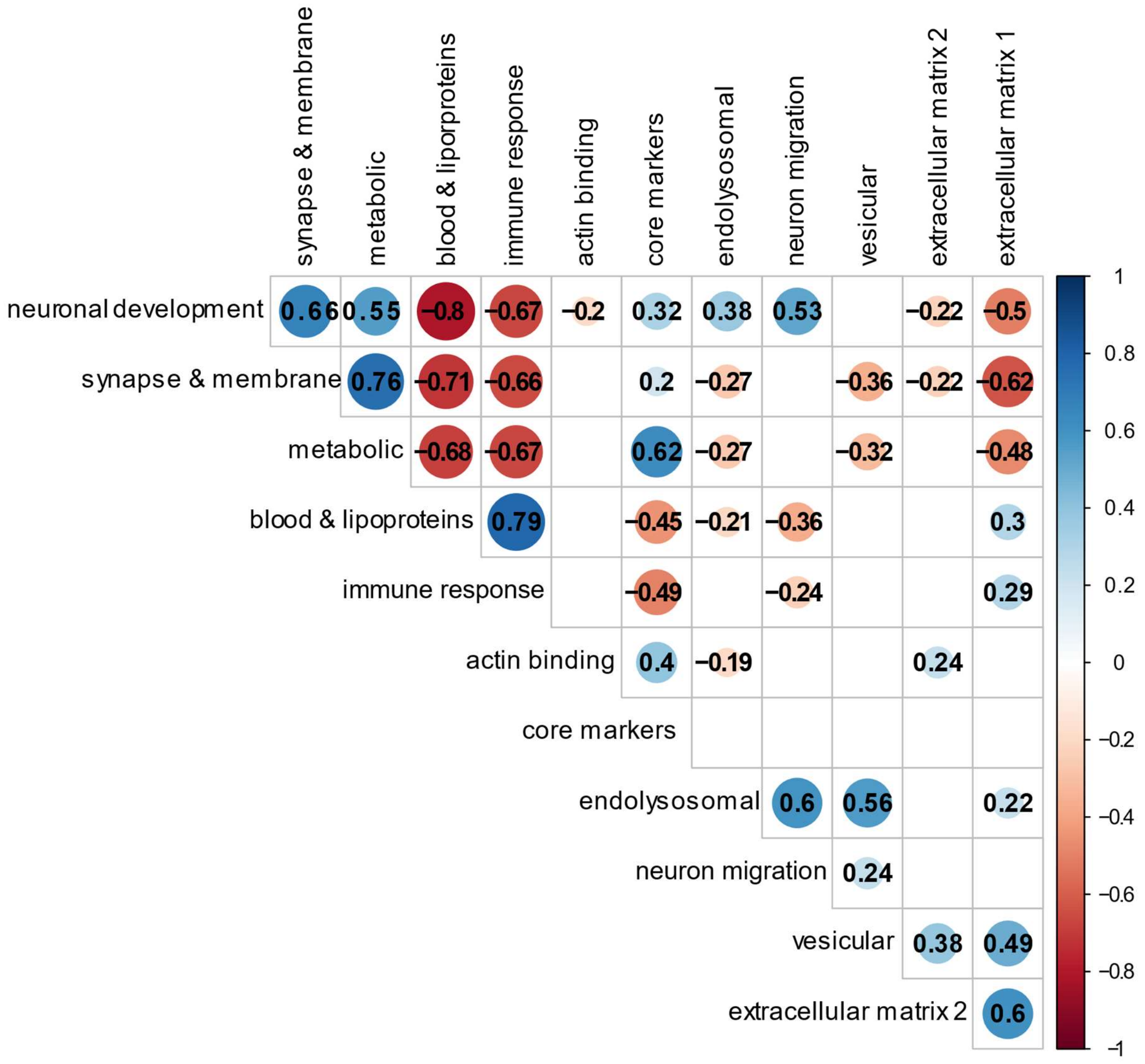

Figure S9. Correlation matrix of cluster Eigenprotein values. Pairwise Spearman rank-order correlation of Eigenprotein values was performed in all participants of the TRIAD cohort. Spearman's ρ of correlations $P<0.05$ are shown.

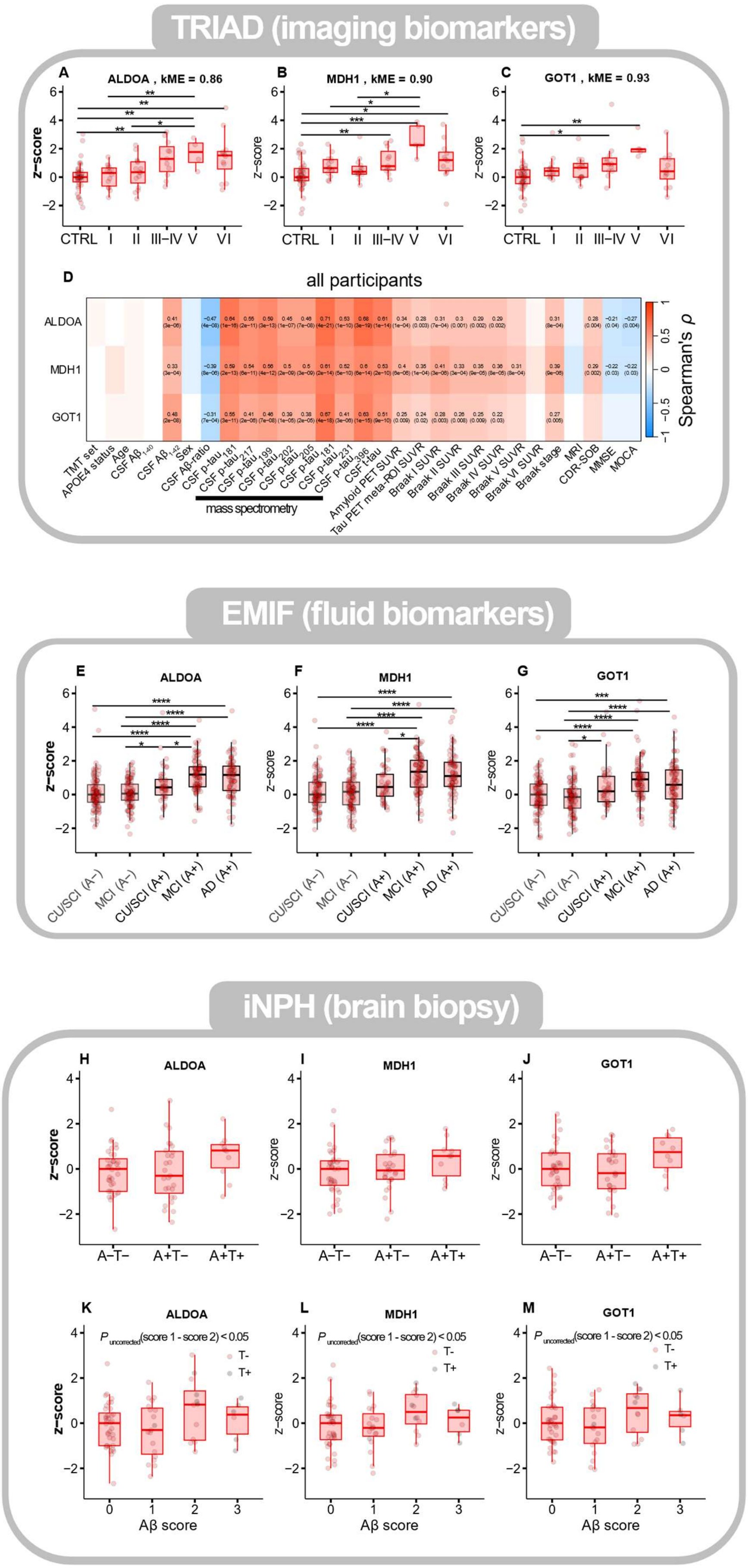

TRIAD (imaging biomarkers)
A
ALDOA , kME = 0.86
B
MDH1 , kME = 0.90
C
GOT1 , kME = 0.93
z−score
CTRL I II III−IV V VI
D
all participants
ALDOA
MDH1
GOT1
Spearman's ρ
TMT set
APOE4 status
Age
CSF Aβ1-40
CSF Aβ1-42
Sex
CSF Aβ-ratio
CSF p-tau181
CSF p-tau217
CSF p-tau199
CSF p-tau202
CSF p-tau205
CSF p-tau181
CSF p-tau231
CSF p-tau396
CSF t-tau
Amyloid PET SUVR
Tau PET meta-ROI SUVR
Braak I SUVR
Braak II SUVR
Braak III SUVR
Braak IV SUVR
Braak V SUVR
Braak VI SUVR
Braak stage
MRI
CDR-SOB
MMSE
MOCA
mass spectrometry
EMIF (fluid biomarkers)
E
ALDOA
F
MDH1
G
GOT1
z−score
CU/SCI (A−)
MCI (A−)
CU/SCI (A+)
MCI (A+)
AD (A+)
iNPH (brain biopsy)
H
ALDOA
I
MDH1
J
GOT1
z−score
A−T−
A+T−
A+T+
K
ALDOA
L
MDH1
M
GOT1
P uncorrected (score 1 - score 2) < 0.05
T-
T+
Aβ score

Figure S10. Boxplots of three constituent proteins of the ‘metabolic’ cluster, ALDOA (A, E, H, K), MDH1 (B, F, I, L), and GOT1 (C, G, J, M) across the AD continuum in the TRIAD (A-C), EMIF (E-G) and iNPH cohort (H-M). (D) Correlation heatmap of relevant parameters of the TRIAD cohort with three constituent proteins of the ‘metabolic’ cluster. (A-C, E-M) Z-scored protein abundances (z-scored to respective reference group) were plotted across clinical groups, stratified by tau PET in the TRIAD cohort (A-C), CSF Aβ status in the EMIF cohort (E-G), and across cortical biopsy groups (H-J) or Aβ scores (K-M), a semiquantitative scale for the Aβ-plaque load in the frontal cortex of patients, in the iNPH cohort. A gray-colored point indicates the presence of neurofibrillary tangles in the cortical sample of the respective individual. Statistical significances for all boxplots were assessed using linear models, with age and sex as covariates, followed by post-hoc Tukey’s HSD for pairwise comparisons to control the family-wise error rate. Unadjusted *P*-values for pairwise comparisons are indicated if relevant. *$P<0.05$, **$P<0.01$, ***$P<0.001$, ****$P<0.0001$. Abbreviations: CU/SCI, cognitively unimpaired/subjective cognitive impairment; MCI, mild cognitive impairment; AD, Alzheimer’s disease; A-, Aβ-negative; A+, Aβ-positive.

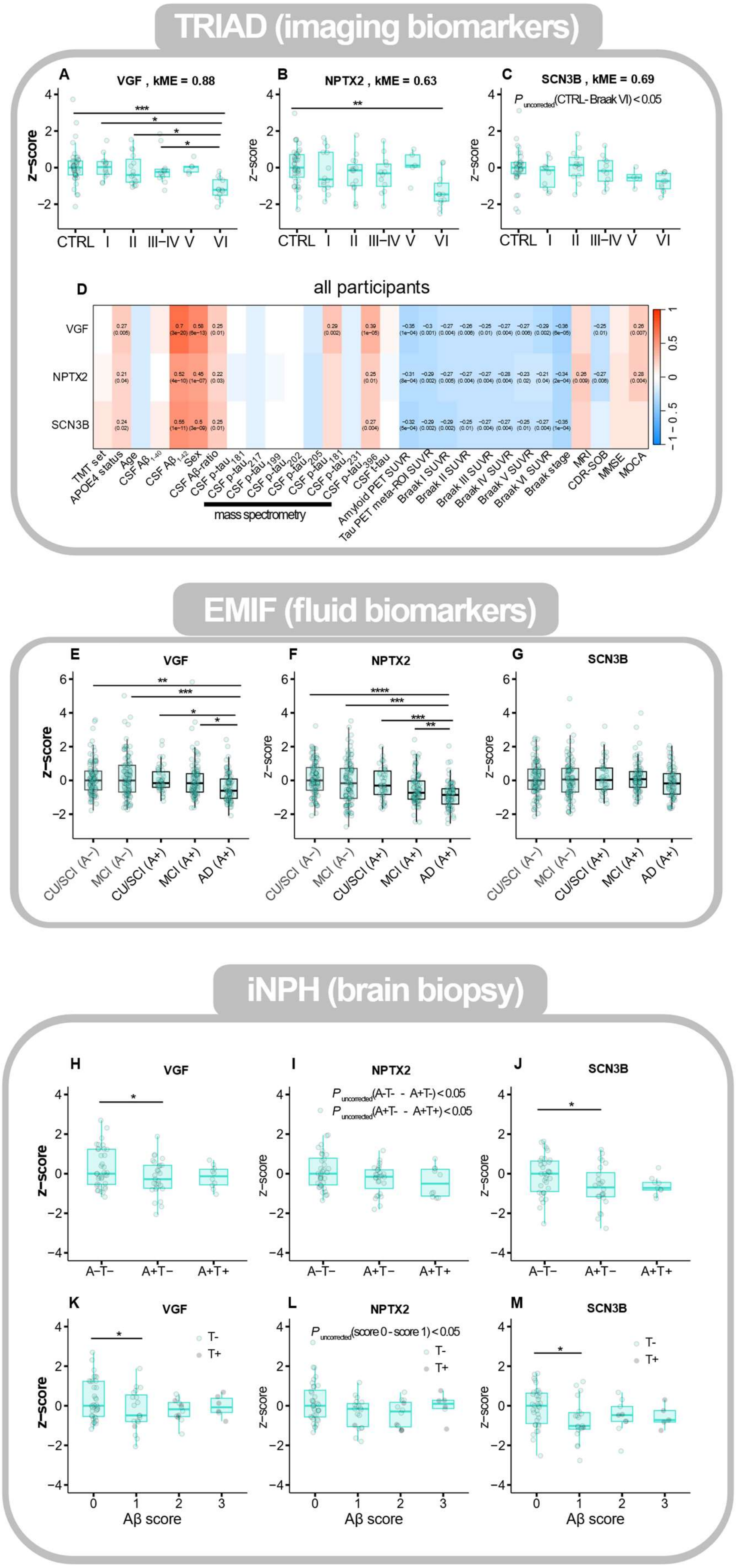
TRIAD (imaging biomarkers)
A
VGF , kME = 0.88
B
NPTX2 , kME = 0.63
C
SCN3B , kME = 0.69
P uncorrected(CTRL- Braak VI) < 0.05
z-score
CTRL I II III–IV V VI
D
all participants
VGF
NPTX2
SCN3B
TMT set
APOE4 status
Age
CSF Aβ1-40
CSF Aβ1-42
Sex
CSF Aβ-ratio
CSF p-tau181
CSF p-tau217
CSF p-tau199
CSF p-tau202
CSF p-tau205
CSF p-tau181
CSF p-tau231
CSF p-tau396
CSF t-tau
Amyloid PET SUVR
Tau PET meta-ROI SUVR
Braak I SUVR
Braak II SUVR
Braak III SUVR
Braak IV SUVR
Braak V SUVR
Braak VI SUVR
Braak stage
MRI
CDR-SOB
MMSE
MOCA
mass spectrometry
EMIF (fluid biomarkers)
E
VGF
F
NPTX2
G
SCN3B
z-score
CU/SCI (A−)
MCI (A−)
CU/SCI (A+)
MCI (A+)
AD (A+)
iNPH (brain biopsy)
H
VGF
I
NPTX2
P uncorrected(A-T- - A+T-) < 0.05
P uncorrected(A+T- - A+T+) < 0.05
J
SCN3B
A−T− A+T− A+T+
K
VGF
L
NPTX2
P uncorrected(score 0 - score 1) < 0.05
M
SCN3B
T-
T+
Aβ score

Figure S11. Boxplots of three constituent proteins of the 'synapse & membrane' cluster, VGF(A, E, H, K), NPTX2 (B, F, I, L), and SCN3B (C, G, J, M) across the AD continuum in the TRIAD (A-C), EMIF (E-G) and iNPH cohort (H-M). (D) Correlation heatmap of relevant parameters of the TRIAD cohort with three constituent proteins of the 'synapse & membrane' cluster. (A-C, E-M) Z-scored protein abundances (z-scored to respective reference group) were plotted across clinical groups, stratified by tau PET in the TRIAD cohort (A-C), CSF Aβ status in the EMIF cohort (E-G), and across cortical biopsy groups (H-J) or Aβ scores (K-M), a semiquantitative scale for the Aβ-plaque load in the frontal cortex of patients, in the iNPH cohort. A gray-colored point indicates the presence of neurofibrillary tangles in the cortical sample of the respective individual. Statistical significances for all boxplots were assessed using linear models, with age and sex as covariates, followed by post-hoc Tukey's HSD for pairwise comparisons to control the family-wise error rate. Unadjusted *P*-values for pairwise comparisons are indicated if relevant. *$P<0.05$, **$P<0.01$, ***$P<0.001$, ****$P<0.0001$. Abbreviations: CU/SCI, cognitively unimpaired/subjective cognitive impairment; MCI, mild cognitive impairment; AD, Alzheimer's disease; A-, Aβ-negative; A+, Aβ-positive.

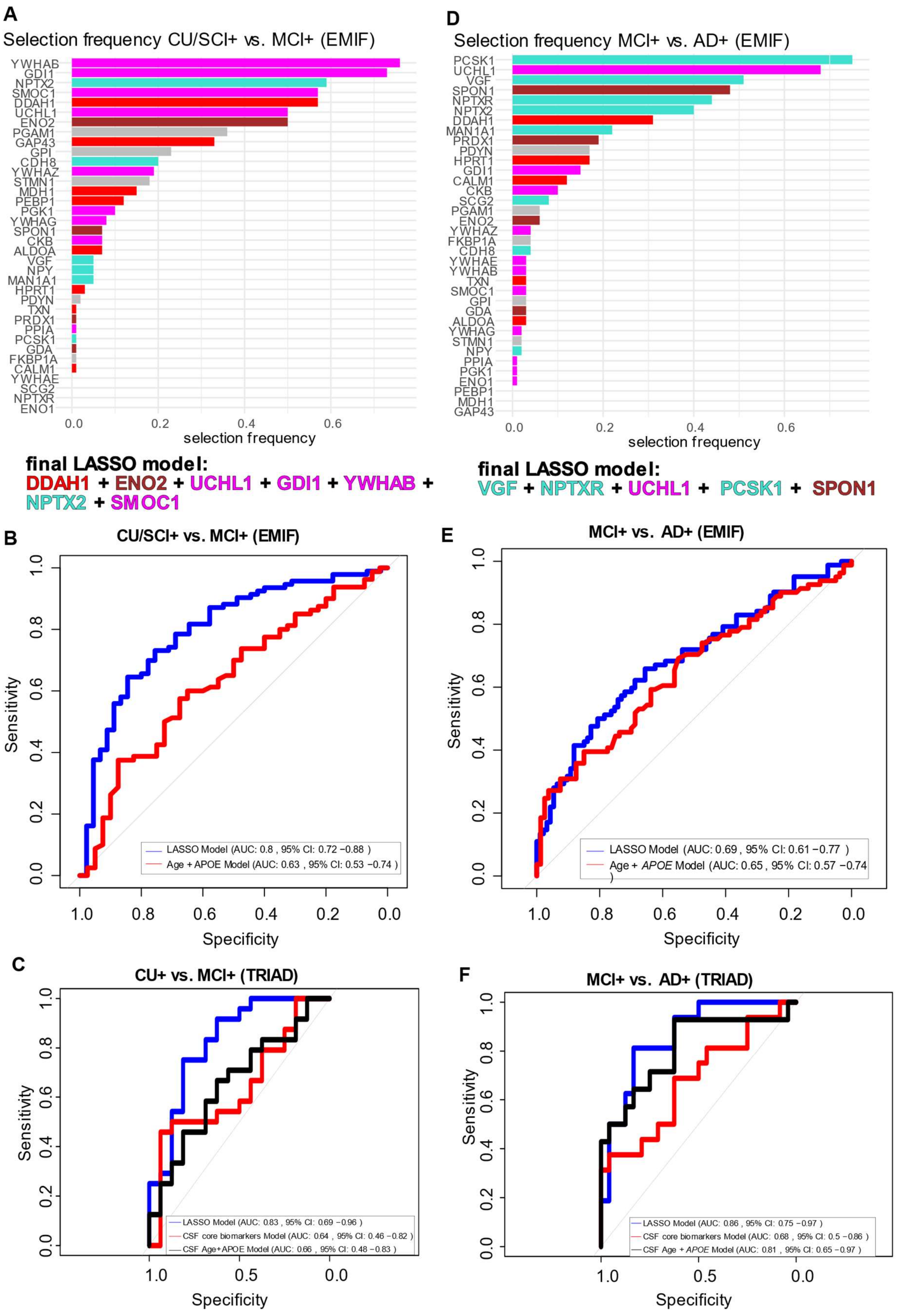

Figure S12. Selection of individual proteins using LASSO regression to distinguish between stages of Alzheimer's disease (AD): preclinical vs. prodromal AD (A–C), and prodromal AD vs. AD dementia (D–F). For LASSO model development, we pre-filtered proteins to include only those that were significantly

different between controls and AD in both the EMIF and TRIAD cohorts (total n = 36), many of which represented hub proteins within the identified protein clusters. LASSO regression was performed in the larger EMIF cohort to identify proteins with predictive value for each group comparison. Bootstrapping (n = 100) was applied to determine the proteins most frequently selected across iterations (A, D). These consistently selected proteins were then used to construct the final LASSO models (A, D). The predictive performance of the final models was subsequently evaluated via ROC analysis in both the EMIF (B, E) and TRIAD (C, F) cohorts.
